# Elevation in tropical sky islands as the common driver in structuring genes and communities of freshwater organisms

**DOI:** 10.1038/s41598-017-16069-y

**Published:** 2017-11-23

**Authors:** Morgan Gueuning, Tomasz Suchan, Sereina Rutschmann, Jean-Luc Gattolliat, Jamsari Jamsari, Al Ihsan Kamil, Camille Pitteloud, Sven Buerki, Michael Balke, Michel Sartori, Nadir Alvarez

**Affiliations:** 1 Department of Ecology and Evolution, University of Lausanne, 1015 Lausanne, Switzerland; 2 Competence Division for Research Technology and Knowledge Exchange, Method Development and Analytics, Agroscope, 8820 Wädenswil Switzerland; 3 Department of Biochemistry, Genetics and Immunology, University of Vigo, 36310 Vigo, Spain; 4 Cantonal Museum of Zoology, Palais de Rumine, 1014 Lausanne, Switzerland; 5 Plant Breeding Section, Faculty of Agriculture, Andalas University, 25163 Padang, West-Sumatera Indonesia; 6 Institute of Terrestrial Ecosystems, ETH Zürich, Switzerland; 7 Swiss Federal Research Institute WSL, Birmensdorf, Switzerland; 8 Department of Life Sciences, Natural History Museum, Cromwell Road, London, SW7 5BD United Kingdom; 9 Department of Biological Sciences, Boise State University, 1910 University Drive, Boise, Idaho 83725 USA; 10 Zoologische Staatssammlung München, Münchhausenstr. 21, 81247 München, Germany; 11 Natural History Museum of Geneva, 1 route de Malagnou, 1208 Geneva, Switzerland; 12 W. Szafer Institute of Botany, Polish Academy of Sciences, ul. Lubicz 46, 31-512 Kraków, Poland

## Abstract

Tropical mountains are usually characterized by a vertically-arranged sequence of ecological belts, which, in contrast to temperate habitats, have remained relatively stable in space across the Quaternary. Such long-lasting patterning of habitats makes them ideal to test the role of environmental pressure in driving ecological and evolutionary processes. Using Sumatran freshwater mayfly communities, we test whether elevation, rather than other spatial factors (i.e. volcanoes, watersheds) structures both species within communities and genes within species. Based on the analysis of 31 mayfly (Ephemeroptera) communities and restriction-site-associated-DNA sequencing in the four most ubiquitous species, we found elevation as the major spatial component structuring both species and genes in the landscape. In other words, similar elevations across different mountains or watersheds harbor more similar species and genes than different elevations within the same mountain or watershed. Tropical elevation gradients characterized by environmental conditions that are both steep and relatively stable seasonally and over geological time scales, are thus responsible for both ecological and genetic differentiation. Our results demonstrate how *in situ* ecological diversification at the micro-evolutionary level might fuel alpha- and beta- components of diversity in tropical sky islands.

## Introduction

Understanding the underlying mechanisms shaping the distribution of biodiversity is a key question in ecology^1^. At a global scale, spatial variation of biodiversity is distributed along two broad axes, latitude and altitude (i.e. elevation)^2^. Whereas the latitudinal biodiversity gradient has been widely studied, the elevational gradient has gained considerable momentum as it provides a framework to investigate biodiversity patterns at a narrower spatial scale^3^. Since the emergence of the species-genetic diversity correlation (SGDC) paradigm^4^, several empirical studies have investigated the parallel between patterns of diversity of species and genes and debated the theoretical grounds of processes producing spatial congruence^5^. Even though SGDC can be skewed by several factors^6^ and therefore create ambiguous conclusions, there is a consensus suggesting a parallel effect of environmental characteristics (area, connectivity, and environmental heterogeneity) on shaping biodiversity at different levels^7^.

However, investigating SGDC does not inform on whether congruence in structuring of species and genes also occurs across space. In other words, are boundaries between different communities and different intra-specific gene pools congruent? Whereas this question has been recently tackled in terrestrial organisms from alpine and temperate environments^8,9^, there has been a lesser effort in tropical macro-invertebrates^10^. Indeed, while spatial patterns of diversity in freshwater macro-invertebrates have been the scope of several studies^11–14^ there is currently a major knowledge gap in our understanding of the relationship between their intra- and inter-specific spatial structuring, especially in tropical regions^15,16^. Here, we use the tropical elevation gradient to test for correlation between boundaries structuring species composition in communities, and those structuring genetic variation within species, using mayflies as a model taxon. Tropical mountains are usually characterized by an elevational succession of a larger number of belts than temperate mountains^17^ and generally show much less overlap in abiotic conditions between low and high elevations than at temperate latitudes^18,19^. As such, a large number of different biotic and abiotic conditions are found along tropical elevation gradients, which are prone to select for a high variety of ecological traits within communities and selection-mediated lineage divergence (if not impeded by gene flow) within species^20^. In addition, whereas Quaternary glacial periods caused dramatic losses in species diversity in high-latitude environments at every glacial maximum, the elevation gradient in the tropics was rather stable climatically through time, and could thus mold biodiversity over the long-term^18,21^.

In this study, we therefore examine the assumption that on tropical mountains, species within communities and genes within species are mainly structured by elevation rather than by other spatial factors, contrasting with the strong imprint of refugia history characterizing higher-latitude communities and species^22^. Because all biotic and abiotic factors shaping ecological niches cannot be accurately measured, we consider the elevation gradient as a surrogate for a wide array of environmental gradients, such as temperature—and in the case of aquatic invertebrates, also pH and stream velocity. The underlying rationale behind our hypothesis is that different habitats found along the elevational succession of ecological belts are ruled by their own physical and biotic parameters, and thus induce differential selection pressures that may filter the species present in communities. In parallel, individuals of a single species distributed along the elevation gradient could adapt to these different selection pressures, and in cases where selection counteracts gene flow along the gradient, differentiation processes might take place^23^. Here, we focus on four Sumatran volcanoes (Indonesia), which we hereafter refer to as sky islands, as an analogy to oceanic islands. We use the aquatic freshwater Ephemeroptera (mayflies) as a model and analyze the population structure of four species among 31 communities—spanning the elevation gradient—to which we apply restriction-site-associated-DNA sequencing^24^ (RAD sequencing). For many aquatic invertebrates the degree of connectivity between populations is determined by the interaction between dispersal traits and the landscape structure^25,26^. Due to their short adult life span, mayflies generally have limited long distance dispersal, which may potentially increase the level of spatial aggregation of species and genes^27^. Furthermore, most mayflies are highly sensitive to biotic and abiotic variations^28^, which makes them ideal model organisms to investigate ecological and evolutionary processes at a narrow spatial scale.

## Results

### Community analyses

**Figure 1:**
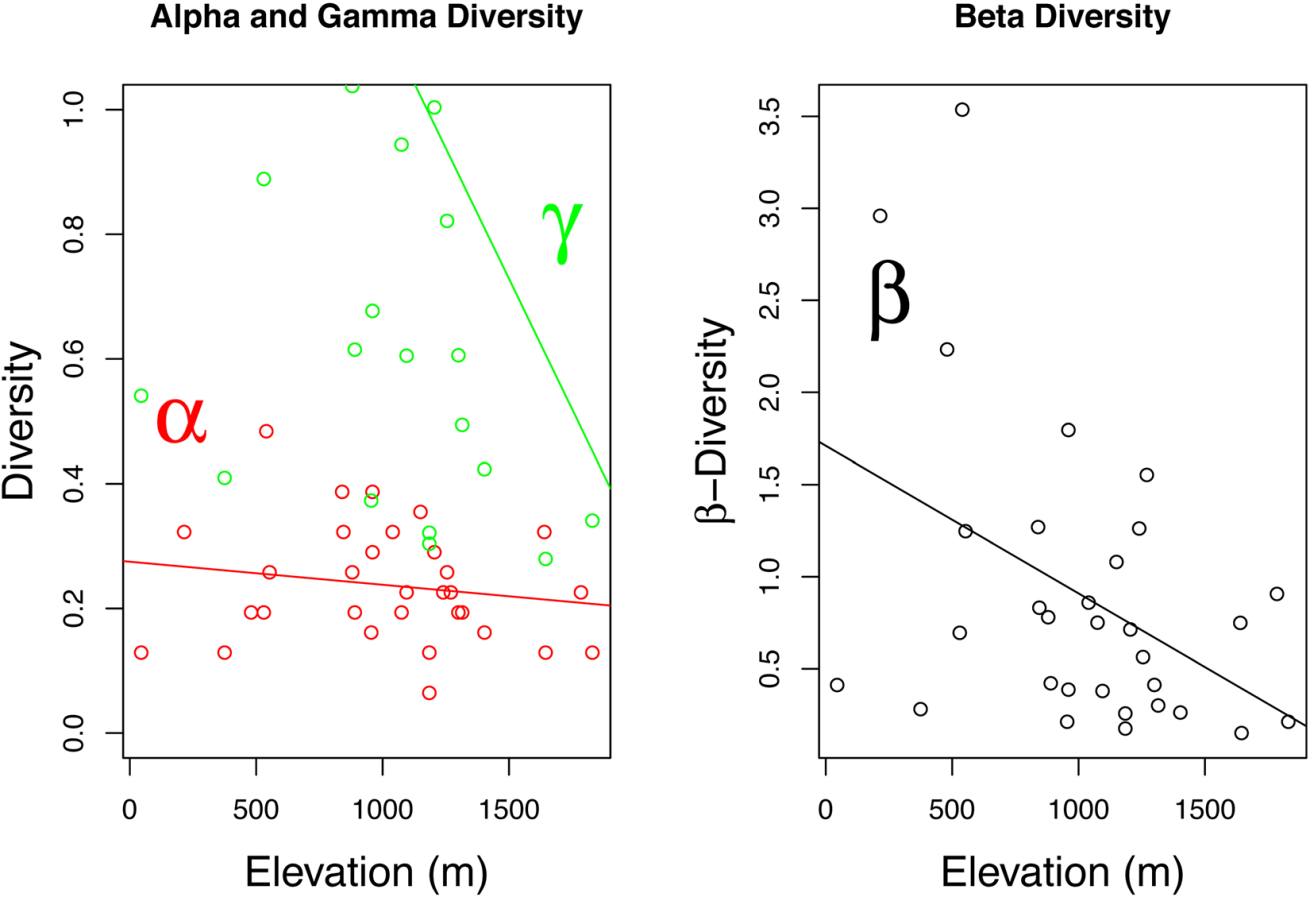
Plotted alpha (α), beta (β) and gamma (ɣ) diversity against elevation. The diversity indexes are estimated by an additive partitioning of species diversity (ɣ = β + α) with gamma being the total diversity, beta the among-site diversity (average amount of diversity not found in a single site), and alpha the average within-site diversity (as described in Lu *et al*.^71^). Linear regressions of observed α, β and ɣ diversity against elevation revealed statistically significant relationships for two indexes, the β and ɣ diversities (β: adjusted R^2^ = 0.1638, *P* = 0.0138; ɣ: adjusted R^2^ = 0.1706, *P* = 0.0121); the correlation between elevation and α diversity was not significant (adjusted R^2^ = 0.0061, *P* = 0.2856).

To examine the relative importance of the elevation gradient compared to spatial features such as watersheds and volcanoes in structuring communities, we performed a non-parametric multivariate analysis of variance using distance matrices computed from the transformed species presence/absence matrix (Supplementary Table S1). The test revealed a highly significant effect (*P* = 0.0001) of elevation on the community structure of mayflies, explaining 15.6% of the variance in species composition across the 31 sampled sites (Supplementary Table S2). To test for spatial autocorrelation in the dataset, we performed a Mantel test between the Euclidean distance matrix among sites and the Bray-Curtis transformed species presence/absence matrix, which uncovered a weak spatial autocorrelation (*R* ^2^ = 0.1132, *P* = 0.0212). Therefore, in order to identify the correlation between community and elevation similarities while controlling for effects of spatial autocorrelation, we conducted a partial Mantel test. The test still revealed a strong correlation between the two matrices (*R* ^2^ = 0.2907, *P* < 0.0001). To further investigate the underlying structure of the correlative relationship between the elevation gradient and the community composition, we used a Mantel correlogram between the Bray-Curtis dissimilarity matrix and the Euclidean matrix of pairwise elevation difference among communities (see Supplementary Fig. S3). The correlogram showed that sampling locations with a difference in elevation of less than 500 meters harbor significantly (*P* < 0.05) similar communities. At the other extreme, sampling locations separated by elevation differences larger than 800 meters display significant differences in community composition (*P* < 0.05). By fitting elevation binary vectors onto the ordination [i.e. by applying a non-parametric multivariate analysis of variance using distance matrices; NMDS] for each 50 meters elevation slice, we identified two elevations, i.e. 850 meters (R^2^ = 0.4424) and 1250 meters (R^2^ = 0.4480), as potential cutoffs best predicting changes in community composition between lowlands and highlands (Table 1). When using species abundance instead of presence/absence, analyses revealed that changes in community composition were better predicted using a cutoff of 850 meters (R^2^ = 0.4798) than 1250 meter (R^2^ = 0.4341). Based on these results and because the 1250 meters threshold lies very near the upper bound of the range for two among the four sequenced species, we fixed the cutoff between lowland and highland at 850 meters. While applying this cutoff to the community data set, all lowland sites encompassed a total of 26 species among which nine were only found at low elevation (below 850 meters). As for highland sites, in total, they encompassed 26 species among which nine were only found at high elevation (above 850 meters). Both highlands and lowlands shared 17 out of the 35 identified species. To test for an elevation gradient pattern in diversity, we computed linear regressions between alpha, beta, gamma diversities and elevation (Fig. 1). The tests revealed statistically significant relationships for two indexes, the beta and gamma diversity (beta: adjusted *R* ^2^ = 0.1578, *P* = 0.01546; gamma: adjusted *R* ^2^ = 0.1451, *P* = 0.01972). Since the linear regression did not reveal a significant relationship between alpha diversity and elevation (adjusted *R* ^*2*^ = −0.004217, *P* = 0.3576), there should be no major bias in sampling size between lowland and highland communities.

**Table 1:** Fitted binary elevation vectors onto non-metric multidimensional scaling (NMDS) ordination of species presence/absence matrix using the Bray-Curtis similarity index.

Elevation class	NMDS1	NMDS2	R2	*P* value
500 m	−0.67523	−0.73761	0.1782	0.072
550 m	−0.67523	−0.73761	0.1782	0.072
600 m	−0.90268	−0.43032	0.3813	0.002
650 m	−0.90268	−0.43032	0.3813	0.002
700 m	−0.90268	−0.43032	0.3813	0.002
750 m	−0.90268	−0.43032	0.3813	0.002
800 m	−0.90268	−0.43032	0.3813	0.002
**850 m**	**−0.90245**	**−0.4308**	**0.4424**	**0.001**
900 m	−0.97197	−0.23509	0.3998	0.001
950 m	−0.97197	−0.23509	0.3998	0.001
1000 m	−0.93194	−0.36261	0.3856	0.001
1050 m	−0.94818	−0.31773	0.3886	0.002
1100 m	−0.84171	−0.53993	0.4012	0.003
1150 m	−0.84171	−0.53993	0.4012	0.003
1200 m	−0.80926	−0.58745	0.4246	0.001
**1250 m**	**−0.70451**	**−0.7097**	**0.4480**	**0.001**
1300 m	−0.72360	−0.69022	0.4177	0.001
1350 m	−0.64759	−0.76199	0.3666	0.002
1400 m	−0.64759	−0.76199	0.3666	0.002
1450 m	−0.67400	−0.73873	0.2786	0.007
1500 m	−0.67400	−0.73873	0.2786	0.007
1550 m	−0.67400	−0.73873	0.2786	0.007
1600 m	−0.67400	−0.73873	0.2786	0.007
1650 m	−0.78487	−0.61966	0.2578	0.013

Elevation was split each 50 meters into binary vectors with “0” encoding for communities sampled below a given threshold and “1” for communities sampled above or exactly at the threshold. Using the Vegan package^82^, we performed a non-metric multidimensional scaling (NMDS) of species presence/absence matrix. By fitting all binary vectors onto the ordination, we retrieved R^2^ values and considered the threshold with the highest R^2^ score as the most meaningful cutoff between lowland and highland. The analysis depicted two maxima with very similar R^2^ values, 850 and 1250 meters above sea level (in bold).

### RADseq analysis

Sequencing of the RADseq library following Mastretta-Yanes *et al*.^24^ produced a total of 295 million reads from 448 individuals in the four—ubiquitous—species spanning the elevation gradient. After *de novo* assembly and quality filtering, including minimum coverage cutoffs and discarding populations represented by single individuals with pyRAD^29^, the number of samples recovered was 84 for *Baetis cf. sabahensis*, 62 for *Bungona* sp., 91 for *Liebebiella cf. vera*, and 62 for *Thalerosphyrus sinuosus*. Reproducibility in the presence/absence of markers was >0.99 in all replicate pairs. Our final dataset comprised between 549 and 1,523 loci, including 4,007 Single Nucleotide Polymorphisms (SNPs) for *T. sinuosus*, 6,052 SNPs for *L. cf. vera*, 8,527 SNPs for *Bungona* sp., and 8,665 SNPs for *B. cf. sabahensis*, characterized by a mean coverage (or depth *sensu* Eaton^29^) of 76, 73, 52, and 26, respectively (pyRAD output files are available on Zenodo, doi:10.5281/zenodo.1045809). In order to produce phylogenetic analyses based on RADseq loci, we distinguished between putative exon and intron loci. In total, we assigned for all four species ≥70% of the loci as introns (*Bungona* sp.: 84%; *L. cf. vera*: 71%; *B. cf. sabahensis*: 82%; *T. sinuosus*: 85%) and ≤20% as exons (*Bungona* sp.: 11%; *L. cf. vera*: 20%; *B. cf. sabahensis*: 12%; *T. sinuosus*: 9%).

#### Spatial genetic structure

**Figure 2:**
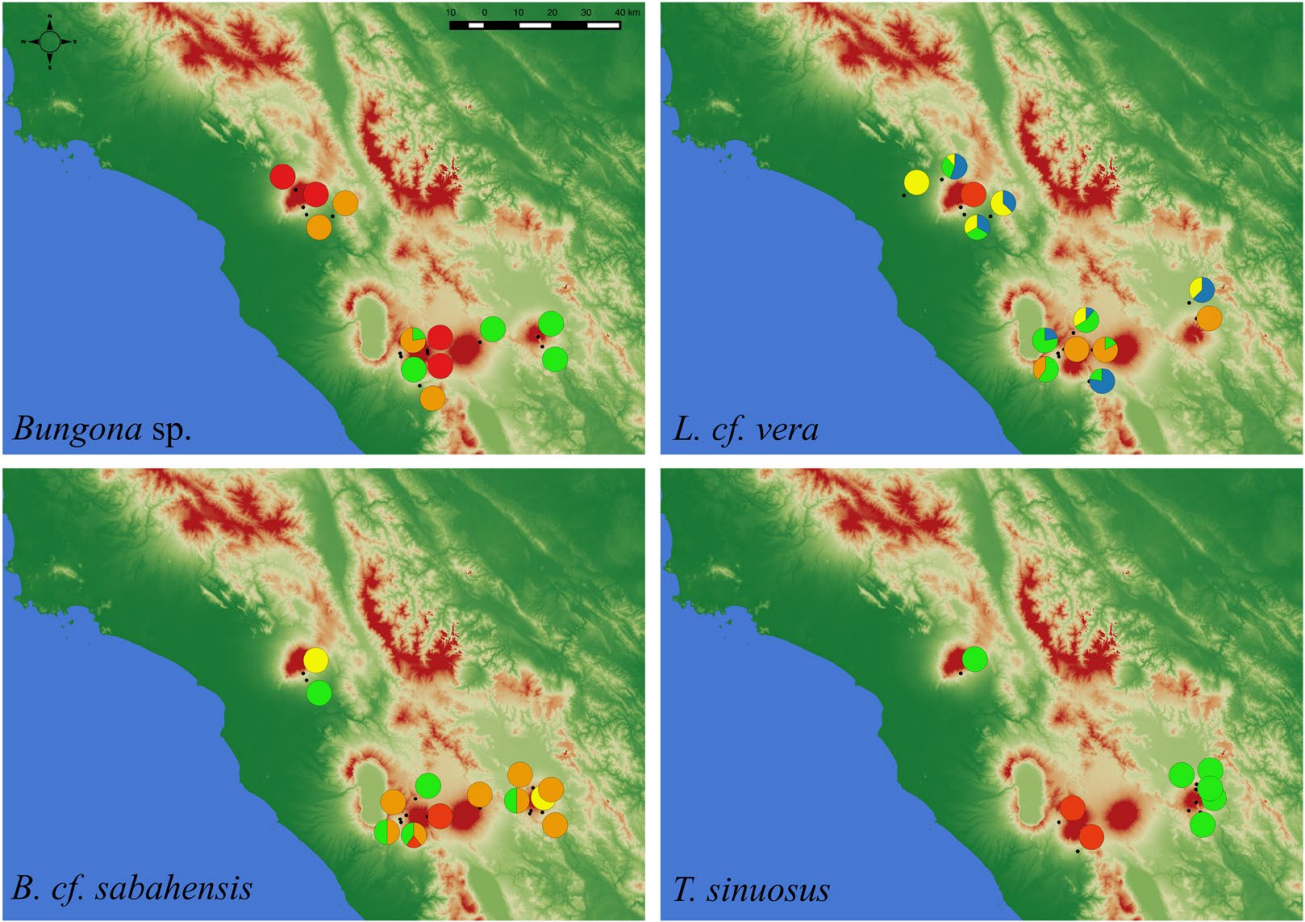
Spatial genetic structure for four Ephemeroptera species showing wide ecological distributions, using restriction-site-associated-DNA sequencing (RADseq). Pie-charts on the map for each species represent the proportion of samples assigned to each cluster within each sampling location. Spatial structure was computed using STRUCTURE, by examining K values ranging from one to eight, and replicating analyses five times. A threshold of 0.95 in the assignment probability was applied to assign samples to a given cluster. The optimal number of genetic clusters was established by inspection of the likelihood function. Visual examination of the distribution of each cluster indicates that populations from a similar elevation tend to be characterized by the same genetic cluster (see also, Supporting Information S3). Maps were generated with the Quantum GIS geographic information system (QGIS 2.18; QGIS Development Team, 2016. QGIS Geographic Information System. Open Source Geospatial Foundation Project; available at https://www.qgis.org).

To investigate the spatial structure of populations in each of the four investigated species we ran Markov chain Monte Carlo (MCMC) analyses, which grouped *Bungona* sp. into three clusters, *L. cf. vera* into five, *B. cf*. *sabahensis* into four, and *T. sinuosus* into two. Spatial genetic structure for each of the four species is depicted in Fig. 2. Based on the spatial distribution of clusters along elevation, a trend towards elevational structuring can be detected in each species with highland and lowland populations, respectively clustering together (Supplementary Fig. S6).

#### Analysis of Molecular Variance

**Figure 3:**
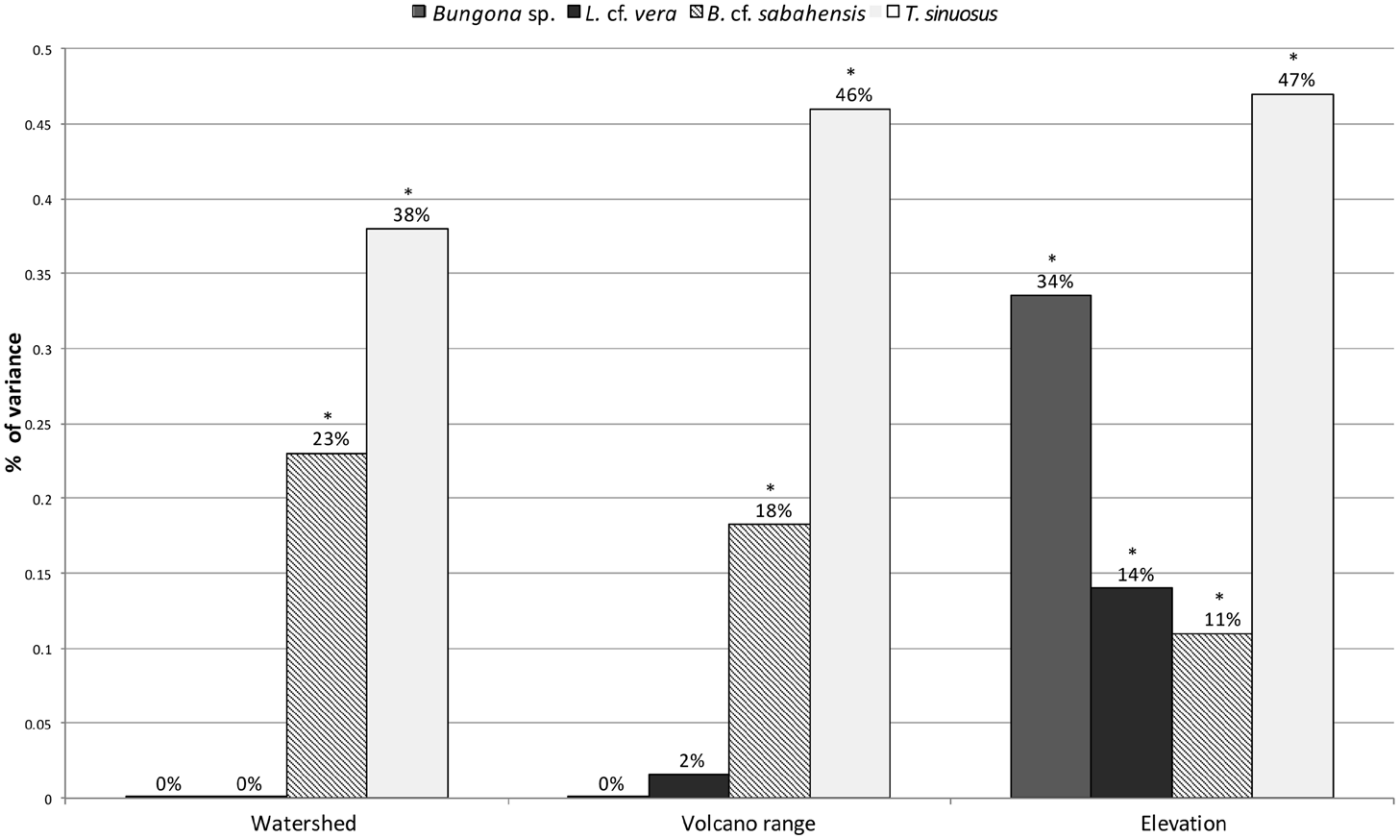
Explained variance at the among-region level using analysis of molecular variance (AMOVA) in four Ephemeroptera species showing wide ecological distributions. Analyses were based on restriction-site-associated-DNA sequencing (RADseq) data. Probability of F_RT_ (i.e., estimated variance among region/total estimated variance) was estimated with 1,000 permutations and *P* values were corrected for multiple-test bias following Bonferroni correction (alpha = 0.05/3 = 0.0167). Asterisks denote significant variance explained by the regional level, characterized by watersheds, volcanoes or elevation.

To partition genetic variance at the regional level we ran analyses of molecular variance (AMOVA), nesting populations into watersheds, volcanoes or elevation (using the 850 meters cutoff based on community composition, see above). Analyses revealed elevation as the only factor that significantly structured the genetic variance across all four species, explaining between 11% (*B. cf. sabahensis*) and 47% (*T. sinuosus*) of the genetic variance (Fig. 3). The structuring effects of watersheds and volcanoes were significant for two among the four species—*B. cf. sabahensis* and *T. sinuosus*. Overall, a relatively high proportion of genetic variation was explained by the among and within population levels (Supplementary Table S7).

When computing species-specific regional differentiation (i.e. F_RT_) between lowland and highland elevation classes, cutoffs at 843, 697, 903 and 898 meters maximized F_RT_ values for *Bungona* sp., *L. cf. vera*, *B. cf. sabahensis*, and *T. sinuosus*, respectively (Supplementary Fig. S8). The four values average at 835 meters, an elevation close to the 850 meters cutoff based on community composition.

#### Isolation by distance

Three Isolation by distance (IBD) analyses per species were performed to determine the relative isolation of populations: (i) for the whole dataset, as well as for (ii) lowland and (iii) highland populations separately. Isolation by distance analyses on combined lowland and highland populations were significant for all species (Table 2). Analyses considering populations in the two elevation categories separately were significant for two species, i.e. *Bungona* sp. and *L. cf. vera*, for both highland and lowland elevation datasets. In these two species, regression slopes were on average 2.9 times steeper for highland than lowland populations. For the two other species, i.e., *B. cf. sabahensis* and *T. sinuosus*, IBD was only significant at high elevation after multiple-test correction.

**Table 2:** Isolation by distance (IBD) results for four Ephemeroptera species showing wide ecological distribution, using restriction-site-associated-DNA sequencing (RADseq).

Types of populations considered	N	Elevation range	R^2^	Regression slope	*P* value	
(a) Lowland and highland populations						
*Bungona* sp.	62	[215 m; 1830m]	0.0908	1.6043x	<0.001*	
*L. cf. vera*	91	[45 m; 1300 m]	0.0220	0.6678x	<0.001*	
*B. cf. sabahensis*	84	[540 m; 1640m]	0.1258	4.2719x	<0.001*	
*T. sinuosus*	62	[375 m; 1255 m]	0.4286	2.4117x	<0.001*	
(b) Lowland populations only						
*Bungona* sp.	30	[215 m; 845 m]	0.0193	0.5394x	0.008*	
*L. cf. vera*	56	[45 m; 845 m]	0.0178	0.5587x	<0.001*	
*B. cf. sabahensis*	18	[540 m; 845 m]	0.0244	12.732x	0.040	
*T. sinuosus*	23	[375 m; 840 m]	0.0129	−0.2142x	0.091	
(c) Highland populations only						
*Bungona* sp.	32	[960 m; 1830m]	0.2812	1.4347x	<0.001*	
*L. cf. vera*	35	[955 m; 1300 m]	0.1050	1.2223x	<0.001*	
*B. cf. sabahensis*	66	[960 m; 1640m]	0.1709	4.9608x	0.010*	
*T. sinuosus*	39	[955 m; 1255 m]	0.05389	0.3631x	0.014*	

IBD values were calculated on linearized F_ST_ and ln(x + 1) transformed geographical distance matrix for (a) lowland and highland populations combined, (b) lowland populations only, (c) highland populations only. Statistical significance was assessed through 10,000 permutations and corrected for multiple-test bias using Bonferroni correction (alpha = 0.05/2 = 0.025).

### Environmental factors

To identify the relative contribution of three recorded environmental factors (i.e. pH, stream velocity and temperature) in structuring genes and communities, we fitted each factor onto the ordination (NMDS) of the species presence/absence matrix as well as the genetic distance matrix of the four analyzed species. Temperature was found to explain both community composition and within-species genetic variation in three species (*Bungona* sp. and *L. cf. vera*, and *B. cf. sabahensis*), despite only few points could be considered due the absence of temperature measurements in most sampling points (Table 3). When excluding temperature from the analyses, and thus reducing the sampling bias, pH and stream velocity were significantly fitted onto the ordinations for four different levels, i.e. community composition and within-species genetic variation in *Bungona* sp., *L. cf. vera*, and *B. cf. sabahensis* (see Supplementary Table S9). Only for one species, *T. sinuosus*, none of the measured factors were found significantly correlated to the ordination axes (Table 3)—this dataset being incidentally characterized by the highest level of missing measurements.

**Table 3:** Results of the environmental factors fitted onto the non-metric multidimensional scaling (NMDS) based on the Bray-Curtis dissimilarity index of the species presence/absence matrix as well as the species genetic distance matrix.

Levels	Data points	Factors	R^2^	NMDS1	NMDS2	*P* value
Communities	14/31	pH	0.9307	0.36577	0.3708	0.098
		Velocity	0.46271	0.88651	0.6852	0.001*
		Temperature	0.99318	0.11658	0.6973	0.001*
*Bungona* sp.	39/62	pH	0.96845	0.24923	0.223	0.013*
		Velocity	0.28892	−0.95735	0.6985	0.001*
		Temperature	0.59737	−0.80197	0.3675	0.001*
*L. cf. vera*	22/91	pH	−0.7019	−0.71228	0.0152	0.859
		Velocity	−0.87667	−0.48109	0.0422	0.659
		Temperature	−0.99639	0.08489	0.9709	0.001*
*B. cf. sabahensis*	53/84	pH	−0.090731	−0.99588	0.1671	0.006*
		Velocity	0.076389	−0.99708	0.333	0.001*
		Temperature	0.022417	−0.99975	0.4599	0.001*
*T.sinuosus*	15/62	pH	−0.15686	−0.98762	0.1128	0.437
		Velocity	0.15686	0.98762	0.1128	0.437
		Temperature	−0.15686	−0.98762	0.1128	0.437

Analyses were performed through 1000 permutations with the vegan package^82^.

**Figure 4:**
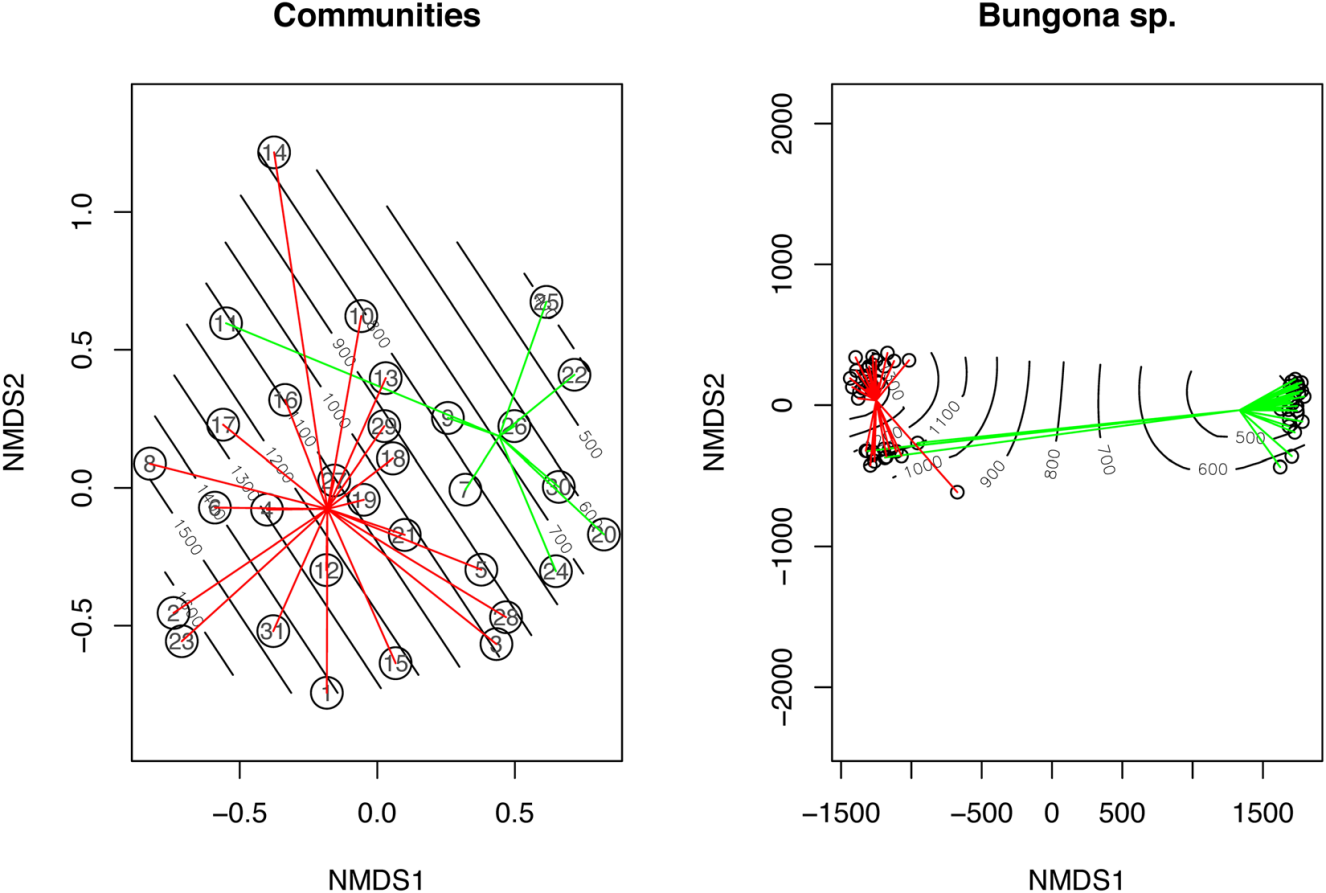
Non-metric multidimensional scaling (NMDS) with stable solution from random starts based on the Bray–Curtis similarity index of the species presence/absence matrix (left frame) and the genetic distance matrix of *Bungona* sp. (right frame; results for the other three species are given in Supplementary Fig. S10). We fitted generalized additive models (GAM) using a 2D smooth surface onto the NMDS site scores. The NMDS analyses were performed with the metaMDS function implemented in the vegan package and isoclines were fitted to the plots with the *ordisurf* function. Furthermore, to visually inspect the accuracy of the models, we added “spider” diagrams connecting communities or individuals from highlands or lowlands to their group centroid.

In addition, to visualize clustering of species within communities and individuals within species along the elevational gradient and to determine how well the ordination fits the elevation factor, we fitted and plotted a generalized additive model (GAM) using a 2D smooth surface onto the NMDS site scores (Fig. 4, Supplementary Fig. S10). In brief, this procedure uses the real multivariate coordinates of populations along the two first explanatory axes, and uses a smoothing algorithm to overimpose elevational isoclines to the multivariate representation.

### Phylogenetic reconstruction

**Figure 5:**
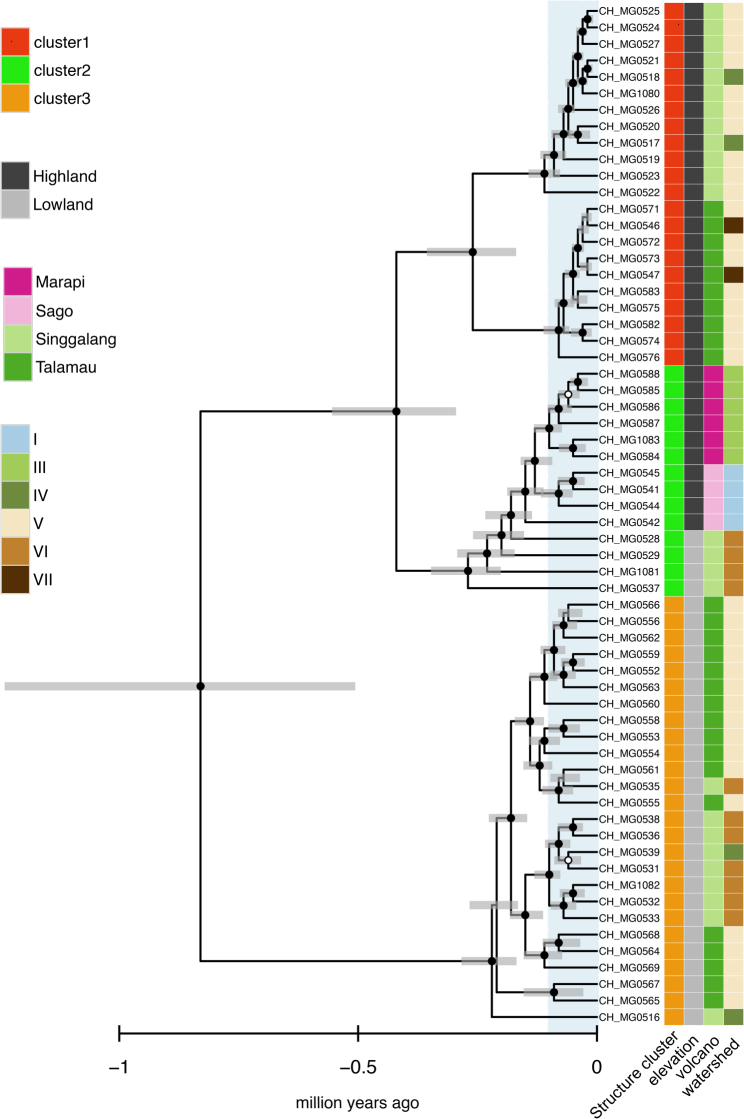
Clock-constrained molecular phylogeny using an *a priori* substitution rate with a Bayesian relaxed clock for the species group *Bungona* sp. (results for the other three species are given in Supplementary Fig. S11). The analyses were based on the concatenated intron data sets. Time axes are given in million years ago (mya) whereby the blue shading corresponds to the last 0.1 mya. Grey bars indicate 95% highest posterior density (HPD) intervals. Black filled circles indicate strongly supported nodes (Bayesian posterior probability (BPP) ≥ 0.95) and white circles moderately supported nodes (BPP ≥ 0.90). Colors to the right of terminal labels indicate the watersheds, elevations, volcanoes, and Structure clusters.

By using an intron-based molecular clock^30^, resulting chronograms show that all four species groups comprised recent speciation events that occurred before three million years ago (mya). We found three supported monophyletic clades for *Bungona* sp. (Fig. 5), six for both *L. cf. vera* and *B. cf. sabahensis*, and two for *T. sinuosus*, with divergence between lowland and highland populations generally occurring within the last million years (Supplementary Fig. S11). Overall, the Bayesian time-calibrated trees and the coalescent-based trees were largely congruent (Supplementary Fig. S12). The Bayesian phylogenetic trees broadly supported the identified Structure clusters (see above). The only exceptions were for one cluster in *L. cf. vera* (cluster 4) and another in *B. cf. sabahensis* (cluster 2), which were both split into two phylogenetic clades.

## Discussion

In this study, we tested the effect of three spatial factors, i.e., elevation, volcanoes and watersheds on the spatial structuring of species within communities and genes within species in tropical freshwater organisms. Based on a survey of 31 mayfly communities, and RADseq genetic analysis of four ubiquitous mayfly species, we found that elevation is the main factor molding biodiversity at both levels.

At the community level, results revealed that elevation is a major factor explaining community composition of mayflies on tropical volcanoes and that similarity in species composition between communities decreases as elevational difference increases (Supplementary Fig. S3). On the ordination plot with the fitted elevation isoclines (Fig. 4), it appears that lowland and highland communities are distinct. Even though the cutoff between both levels is not absolute, there is a clear shift in community composition with only ca. 48.6% (17/35) of shared species between lowland and highland communities. Similar patterns of species turnover were found by Monaghan and colleagues^31^ with sites at different elevations often sharing less than 50% of taxa. Another study examining the influence of environmental and spatial processes in shaping tropical mayfly communities^32^ found that both elevation and watersheds influenced the communities composition, the latter being explained by high-levels of between-basins connectivity, which should facilitate species dispersal by larval drift downstream and by nuptial flight upstream^33,34^. Interestingly, Shimano and colleagues^32^ found a significant effect of spatial factors on community composition when using the species abundance matrix and not the species occurrence matrix, suggesting a more rapid change in species abundance than occurrence towards environmental variations. As predicted by the unified neutral theory of biodiversity^35^, similarity in species composition between communities decreases with distance. Nonetheless, the observed pattern of dissimilarity induced by elevation when controlling for distance is not compatible with the neutral theory, and suggests that other ecological processes are molding community composition. Furthermore, contrasting with patterns found along the latitudinal gradient, we found no evidence of decrease in species richness (alpha diversity) towards higher elevations (Fig. 1). Even though the global pattern of diversity variation along the elevation gradient in tropical regions is generally unimodal with prediction of species richness decreasing towards higher elevations, this pattern can highly vary among taxa^36^ or study scales^37^. Nevertheless, within the niche theory framework, a decrease in species richness would be linked to the decrease in available conditions at each site. In our data set, the lack of decrease in species richness and the high turnover rate along the elevation gradient suggests a steep shift, instead of a decrease, in ecological (abiotic) conditions^38,39^. Because ecological transitions along the elevation gradient of sky islands occur at exceptionally small scales in the tropics^19,40^, each transition should be associated with changes in key parameters shaping the ecological niches and thus, the community structure along the gradient. Our results are therefore in agreement with the niche theory^41^, in the sense that similar environments should be associated with similar species composition^42^. Congruent with our results, studies focusing on the macro-zoobenthos community composition found similar patterns with elevation acting as an important factor in determining taxonomical composition^11,43–46^.

At the intra-specific level, the model-based spatial genetic structure analysis of within-species genetic variation as revealed by RADseq demonstrated a trend towards elevational structuring with highland populations—and lowland populations, respectively—grouping in different clusters (Fig. 2, Supplementary Fig. S6). This result was also illustrated by phylogenetic analyses, showing that the divergence between low- and high-elevation populations usually originated shortly before or during the last million years (Fig. 5, Supplementary Fig. S11), a time frame compatible with ongoing speciation. The AMOVA confirmed the role played by elevation: despite the geographical distances separating the four volcanoes and the high connectivity expected within watersheds, elevation was the only factor that significantly explained molecular variance across all four species (Fig. 3, Supplementary Table S7). This pattern finds an explanation in the wide environmental changes experienced by aquatic and terrestrial communities along the elevation gradient. As described above, tropical sky islands are characterized by strong abiotic and biotic gradients along the successive ecological belts, which impose selective pressure on species with narrow ecological tolerance^20,47^. Furthermore, higher speciation rates are expected to occur in stable habitats because of reduced dispersal^48^—as it is the case in tropical biomes, which were less affected by Quaternary climatic oscillations than, for instance, temperate habitats.

Divergent selection pressures across ecological belts may produce local adaptation and eventually lead to speciation if selection is strong enough to counteract gene flow^47,49,50^. Several empirical studies provide evidence of ecological speciation along gradients in freshwater organisms. For instance, Vuataz and colleagues^51^ investigated speciation and associated mechanisms along the elevational zonation of alpine streams. Surprisingly, they found a constant speciation rate, which could not be caused by paleoclimatic fluctuations, but rather by a trend towards downstream diversification as predicted by the headwater origin hypothesis^52,53^. For *Bungona* sp. and *L. cf. vera*, in which elevation was the only factor found to be driving within-species genetic structuring, this model—referred to as the gradient model by Moritz and colleagues^47^—applies well. It also partly applies to *B. cf. sabahensis* and *T. sinuosus*, although these species further experience lineage divergence due to spatial isolation mediated by volcanoes and watersheds. While Sumatran volcanoes are characterized by centurial eruptive events and landslides, which can potentially affect habitat availability, such dramatic events almost never affect more than one volcano at a time. As a result, local extinction should be followed by recolonization from populations at similar elevations on neighboring volcanoes, a scenario in agreement with our findings.

Isolation by distance analyses conducted using populations at high and low elevation separately, revealed greater isolation by distance among highland populations than among those in the lowlands (Table 2). This is congruent with the prediction that high-elevation habitats on sky islands experience less gene flow and more drift than populations in the lowlands, a pattern that has also been detected in other aquatic insects^15,54,55^. Dispersal among highland populations of different volcano ranges is indeed limited, as it may require passing through ecologically sub-optimal lower-elevation belts. Isolation of highland populations is known to be an important trigger for diversification and is one of the key factors explaining the high level of endemism found on sky islands^56^. In freshwater systems, headwaters are known to be an important source of macroinvertebrate diversity in stream networks^39^. Not only they harbor headwater specialists, but they also play an important role as refugia during periods of extreme biotic (e.g. predators and invasive species) and abiotic variation. Even though in most cases, patterns of macroinvertebrates along longitudinal gradients follow a humped-shaped pattern with the mid-order streams harboring the highest richness^57^, headwaters are thought to contribute to a high extent to the diversity of river networks^58^. At the genetic scale, headwater specialists are often highly isolated because of more reduced ability towards between-stream dispersal compared to taxa inhabiting low-elevation environments^54^, although this pattern highly depends on intrinsic dispersal capacities^59^. At the scale of our study, we think that dispersal of adults is not impeded by such limitations and could allow gene flow among highland populations located on different volcanoes and in different watersheds.

Results from among- and within-species analyses showed similarities in terms of spatial structuring. Both levels were mainly structured by elevation, a result highlighting the importance of habitat selection for shaping species and gene assemblies, especially in temporally stable environments^60^. Even though the common denominator for structuring communities and genes is elevation, the magnitude at which this factor shapes the two levels is not equal. Whereas elevation was the only component shaping the community structure in our data set, additional factors also influenced the genetic structuring in half of the investigated taxa. Indeed, our results for *B. cf. sabahensis* and *T. sinuosus* showed that spatial components such as volcanoes and watersheds were also key in explaining gene-flow—one might thus consider that both geographical variation in topological features and species dispersal capacities are good predictors of gene flow^61^. Nevertheless, the major importance of elevation in explaining within-species genetic variation in all four investigated species is even more remarkable here due to the fact that lowland and highland populations are much closer geographically compared to populations located on different volcanoes or watersheds —and thus less prone to show spatial effects simply due to patterns of isolation by distance.

If elevation has been shown to be a crucial factor in determining community composition and acting as gene flow barrier at the intra-specific level, the mechanisms behind such a pattern remain however complex. Being a composite variable of numerous ecological and spatial factors, its ability to shape biodiversity at species and gene levels is due to the combination of all underlying factors correlated with elevation. At the community level for instance, abiotic parameters correlated to elevation such as water temperature, pH, stream velocity (also recorded in this study; Supplementary Fig. S13), stream size, oxygen concentration, and contamination level, as well as biotic (riparian conditions, predators’ community type) parameters have been found to play a significant role in the assemblage of macroinvertebrates^62–69^. Prone to vary along the elevation gradient, each of these parameters potentially contributes to the definition of a species’ niche and may drive disruptive selection at the intra-specific level. To investigate which and the extent to which proximal abiotic environmental factors varying along the elevational gradient were shaping communities and genes in our data sets, we partitioned the variance into three factors, i.e pH, temperature and velocity (Table 3). Even though results of these analyses have to be taken with caution because of the relatively high number of missing data and the fact that the measured abiotic factors are largely confounded along the elevation gradient, our results suggest similar effects of the recorded environmental factors on the structuring of communities and genes. Indeed, for instance, temperature was found to be significantly correlated to the structuring of species within communities as well as of genes within species for three out of the four species, a result in agreement with Jacobsen and colleagues^63^ for the community level, but adding evidence for selection at the microhabitat scale for the intra-specific level.

One of the major concerns when investigating diversity at the community or genetic level, is the assessment of cryptic species. The presence of cryptic species can lead to an under evaluation of biodiversity and more importantly to erroneous inference on genetic diversity^70^. To account for cryptic lineages in morphospecies, or resolve recent diversification events, Rutschmann *et al*.^71^ suggested an approach combining coalescent-based phylogeographic, species delimitation and phylogenetic reconstruction using a large number of nuclear genes. Even though no species delimitation essay was performed in the present study, the coalescent-based phylogenetic reconstruction shows that ongoing divergence between low and high elevation populations generally dates back from less than a million years ago, a scale that is compatible with the intra-specific evolutionary time scale. The majority of molecular studies on endemic species indicate that Pleistocene climate oscillations only had a minor role in driving lineage divergence in tropical species^47^, although several case studies are challenging this paradigm^72–74^.

In conclusion, our study provides evidence of the importance of elevation in structuring species and genes in the aquatic ecosystems of tropical sky islands. We propose that the ecological and genetic barrier between lowlands and highlands is induced by strongly contrasted but temporally-stable conditions along the elevation gradient of tropical volcanoes. This framework promotes differential ecological filtering or competing interactions^75^ as well as diversifying natural selection at a small spatial scale, which have implications at the macro-ecological scale. Elevation gradients should thus be considered as an essential ecological and evolutionary component molding biodiversity. Furthermore, our study contributes to the broadening of the debate on the SGDC paradigm by providing important evidence of the key role of environmental characteristics in shaping genes and species in parallel. Eventually, we demonstrate how *in situ* ecological diversification at the micro-evolutionary level is potentially fueling alpha- and beta-components of diversity in tropical sky islands. Because our conclusions provide additional evidence of the importance of the interaction between evolutionary and ecological processes in shaping biodiversity^76^, they also bring a new insight into our understanding of the patterns of high levels of species diversity often found in oceanic islands^77^. Here, we advocate that steep elevation gradients characterizing a large number of oceanic islands are driving high levels of species diversity through micro-evolutionary processes such as those highlighted in the current study.

Finally, if our study provides empirical evidence for structuring genes and communities along the elevational gradient in tropical freshwater organisms, more research is needed to understand the extent to which the elevation gradient acts as a global biodiversity shaping factor.

## Methods

We sampled 31 mayfly communities along the elevation gradient of four Sumatran volcanoes, and performed restriction-site-associated-DNA sequencing [RADseq^24^] on the four most ubiquitous species, in order to test the hypothesis that elevation structures both species within communities and genes within species.

### Study area

Sampling took place in Spring and Summer 2014. The study area was located in West Sumatra (Indonesia), a volcanic region hosting several isolated volcanoes. We focused on four volcanoes—Mt Marapi (2,891 m), Sago (2,271 m), Singgalang (2,877 m), and Talamau (2,912 m)—which display similarities in terms of geology, topography and hydrology, with at least one shared watershed between volcanoes (Supplementary Fig. S14). Each volcano is classified as a nature reserve and very little anthropogenic intervention was observed; however, volcanoes were separated by intensively managed and urbanized valleys. The volcanoes are separated by distances of between 14 and 90 km.

### Sampling

On each volcano, we sampled one to two streams per watershed (Supplementary Fig. S14, Supplementary Table S15), summing up to 16 streams in total. Within each stream we sampled at lower and upper stream sections, with the exception of three unreachable upper sections replaced by two extra samplings in one stream, yielding a total of 31 sampling sites. Sampling sites were chosen based on high levels of micro-habitat variability and on their accessibility. They were circumscribed as ten times the mean stream width at a given location. Within each sampling site, larvae were sampled with standardized Surber nets at three points and side points were sampled with hand nets. Specimens were directly preserved in 99% EtOH on site and later stored at −20 °C in refreshed EtOH until DNA extraction.

### Species identification

All sampled individuals (2,384) were sorted by species/morphospecies, based on morphological characters [following Müller-Liebenau^78^ and Sartori^79^], or morphospecies for non-described species. In total we identified 35 species or morphospecies belonging to nine families.

### Community data analyses

Because time allocated for sampling was not equal among all sites, we used the species presence/absence matrix as a measure of community composition. To examine the relative importance of elevation, watersheds and volcanoes in structuring communities, we performed a non-parametric multivariate analysis of variance using distance matrices [i.e., permutational manova^80^]. We used the *adonis* function in the R CRAN^81^ vegan package^82^, which we parameterized with 10,000 permutations using the Bray-Curtis dissimilarity measure applied to the species presence/absence matrix. Dissimilarity matrices for all spatial features investigated were produced using (i) Euclidean pairwise metric distances among communities for elevation and (ii) binary matrices for sharing/not sharing of watershed or volcano among communities. To take into consideration spatial autocorrelation, we performed a Mantel test between geographical distance and the community composition distance matrix. Since the data showed spatial autocorrelation (see Results section), we computed a partial Mantel test to assess the relative importance of elevation on community composition while controlling for the effects of geographic distance. We used 10,000 permutations for both the Mantel and partial Mantel tests to assess statistical significance. The Mantel correlogram was computed between the Bray-Curtis composition dissimilarity matrix and the Euclidean matrix of pairwise elevation difference among communities, as implemented in the vegan package. Finally, to understand how communities are structured along the elevational gradient and identify differences in species composition turnover among sites, we plotted the observed alpha (α), beta (β), and gamma (ɣ) diversity estimators against elevation. We used a derived method that quantifies the species and sites distinctiveness and attributes the additive beta diversity component to each site^83^. The diversity indexes are estimated by an additive partitioning of species diversity (ɣ = β + α) with gamma being the total diversity, beta the among-site diversity (average amount of diversity not found in a single site), and alpha the average within-site diversity (for computational details, see Lu *et al*.^83^). Indexes were computed by the *contribdiv* function of the vegan package using the species richness index. To test for elevation gradients in diversity, we regressed the observed alpha, beta and gamma diversities against elevation using linear regressions.

In order to identify the elevation cutoff between lowlands and highlands that best predicts changes in community composition, we performed a non-metric multidimensional scaling (NMDS)—based on the Bray-Curtis similarity index—of the species presence/absence matrix^84^, using the vegan package. Elevation was split each 50 meters into binary vectors, encoding for communities sampled below a given threshold and for communities sampled above or exactly at the threshold. By fitting all binary vectors onto the ordination, we retrieved R^2^ values for each threshold and considered the value with the highest R^2^ score as the most meaningful cutoff between lowlands and highlands. Because two elevation cutoffs were associated with nearly identical maximum R^2^ values (see Results section), we performed the same analytical procedure using the species abundance matrix to discriminate between the two.

### RAD sequencing

Based on their ubiquitous distribution at low and high elevation in our dataset, four species (*Bungona* sp., *Liebebiella. cf. vera*, *Baetis cf. sabahensis* and *Thalerosphyrus sinuosus*) were selected for genetic analysis and prepared with a double digest RAD library. For each species, ca. ten individuals per population were sequenced. Samples were digested overnight with a proteinase K solution at 56 °C, allowing the preservation of the exoskeleton for microscopic mountings. DNA extractions were performed using a Biosprint 96 extraction robot (Qiagen, Hombrechtikon, Switzerland), following the manufacturer’s protocol.

Double digestion was performed using the restriction enzymes SbfI and MseI. The protocol (see Supplementary Protocol S16) was slightly modified from that of Mastretta-Yanes and colleagues^24^. In total, we used 96 uniquely barcoded adaptors and five Illumina indexes to build a RAD library incorporating in total DNA of 448 individuals across the four species. To minimize plate effects, samples were assigned semi-randomly to PCR plates so that each plate contained at least one individual from each population and species. In addition, each plate included two blanks and six randomly chosen replicates for inter-plate comparisons and reproducibility rate estimation. The library was sequenced twice using a single-read protocol on an Illumina 2500 HiSeq (Lausanne Genomic Technologies Facility, University of Lausanne, Switzerland).

### RADseq output treatment

We used the pyRAD v.3.0^29^ pipeline to demultiplex, filter and assemble the RADseq outputs. The software pyRAD allows *de novo* clustering of reads from different individuals into loci and further SNP calling. It has the advantage over other pipelines [e.g., Catchen *et al*.^81^] of better dealing with indel containing loci and incompletely overlapping reads^29^. Minimum sequencing depth (Mindepth) was fixed to six and the clustering threshold value for Vsearch^85^ set to 0.88. Additionally, we filtered out reads with more than four sites per read with an error rate >1% and set the maximum proportion of shared polymorphic sites in a locus (MaxSH) to three. Other parameters were left to default values. After retrieving filtered sequences from each individual, we applied a species-specific cutoff to discard individuals and loci with low coverage (i.e., less than 10% representation). For the time-calibrated phylogenetic analysis (see below), we filtered out the RADloci being assigned as intron regions. Therefore, we used the longest sequence of each RAD locus for blastx searches^86^ against the insect protein database (“txid50557 [Organism:exp]”) and used an e-value threshold of 1e-2.

### Spatial genetic structure

We used STRUCTURE v.2.3.4^87^ to investigate the spatial structure of populations in each of the four species. We ran MCMC analyses with 200,000 burnin cycles and 1,000,000 sampled cycles using the “no admixture” and “independent allele frequencies” parameters. For each species we examined K values ranging from one to eight, and replicated each analysis five times. Optimal K values were determined by plotting the highest “estimated Ln probability of the data” among the five replicates of a given K value, against K. The statistical criterion (≥0.95 in the assignment probability) was applied to assign samples to a given cluster. Cluster assignments were displayed on geographical maps using QGIS (QGIS Development Team), by representing each population as a pie-chart showing the number of samples assigned to each cluster. In order to depict spatial distribution of genetic clusters along elevation, pie-charts were also plotted as a function of elevation, irrespective of volcanoes and watersheds.

### Analysis of Molecular Variance

To partition genetic variance at the regional level we performed an analysis of molecular variance (AMOVA) using GenAIEx v.6501^88^. This was done to test the structuring effect of spatial components: volcanoes (maximum four categories), watersheds (maximum six categories) and elevation (two categories—lowland vs. highland). Populations represented by only one individual were discarded from the analyses. Probability of F_RT_ (i.e. estimated variance among region/total estimated variance) was assessed using 1,000 permutations and *P-values* were corrected for multiple tests using Bonferroni correction. GenAlEx was also used to investigate whether populations were genetically isolated by distance (IBD). IBD analyses were performed using a Mantel test with 10,000 permutations, computing the correlation between linearized F_ST_ (i.e. estimated variance among region and populations/total estimated variance) and *ln*-transformed geographical distance matrices. Because each observation was used to compute IBD both at the overall and highland or lowland levels, we applied a Bonferroni multiple test correction and considered significance using alpha = 0.05/2 = 0.025. In order to determine if the cutoff based on the community composition (see above) was consistent with the cutoff observed at the intra-specific level, we also identified species-specific cutoffs by computing F_RT_ scores between lowland and highland elevation classes among all possible dividing points and considered the threshold value that maximized F_RT_. In order to ensure at least two populations per elevation class, we selected the second highest F_RT_ value in cases where the highest F_RT_ value fell at one end of the elevation gradient. The cutoff points were computed as the median values between two successive elevational sampling stations.

### Environmental factors

Three recorded environmental factors (i.e pH, stream velocity and temperature) were fitted onto the ordination (NMDS) of the species presence/absence matrix as well as the genetic distance matrix of the four sequenced species using the vegan package. Community and genetic distance matrices were transformed using the Bray-Curtis dissimilarity index and fitting of the environmental factors was done through 1,000 permutations with the function *envfit* of the vegan package. Because the temperature variable could not be measured in 17 of 31 sampling points, fitting this variable considerably decreased the data size and thus increased possible biases. Therefore, for each data set, we performed the analyses with and without temperature, and accounted for multiple tests correction as described above. To visually evaluate the accuracy of the model, we added a “spider” diagram onto the ordination plot connecting communities or individuals from highlands or lowlands to their group centroid.

### Phylogenetic reconstruction

Time-calibrated Bayesian phylogenies for each species were estimated using BEAST v.2.4^89^. To date diversification events within each species, we used previously published insect mutation rates estimated from arthropods^30^. We applied an intron-specific mutation rate of 3.68% divergence per million years^30^. The phylogenetic trees were calculated separately based on the concatenated intron alignment. First, we used the GTR + Γ model of evolution. Since these specifications yielded a poor sampling of several parameters [effective sample sizes (ESS) < 50], we additionally used a HKY + Γ model. A Bayesian relaxed (uncorrelated lognormal) clock with a constant-size coalescent was used. We ran multiple independent runs with 10^9^ generations, sampling trees every 10^4^ generations. For subsequent analyses, we selected among all ten runs, the one with the best convergence (ESS >100) assessed using Tracer v.1.6^90^. Maximum clade credibility trees were obtained using TreeAnnotator v.2.4^89^, whereby we discarded 10% as burnin.

The phylogenetic relationships within each species were additionally estimated using a quartet-based coalescent-approach. For this we used the program SVDquartets^91^ within PAUP* v.4.0a149^92^. We evaluated all quartets and ran 1,000 bootstrap replicates. All trees were plotted with the R package ggtree^93^.

## Electronic supplementary material

Supplementary material S1–S16
