# Supplementary material for: Elevation in tropical sky islands as the common driver in structuring genes and communities of freshwater organisms
Source: Sci Rep. 2017 Nov 23;7:16089. doi: 10.1038/s41598-017-16069-y (PMC5700956; doi:10.1038/s41598-017-16069-y)
Supplement: Supplementary file 1 — Supplementary material S1–S16 [file 41598_2017_16069_MOESM1_ESM.doc]

**Elevation in tropical sky islands as the common driver in structuring genes and communities of freshwater organisms**

Authors: **Morgan Gueuning, Tomasz Suchan, Sereina Rutschmann, Jean Luc Gattolliat, Jamsari Jamsari, Al Ihsan Kamil, Camille Pitteloud, Sven Buerki, Michael Balke, Michel Sartori & Nadir Alvarez**

**Supplementary material**

**S1 Abundance matrix for the 35 collected species across the 31 sampling sites.**

| **Family** | **Species** | **Sin.R1.L1** | **Sin.R1.L2** | **Sin.R2.L1** | **Sin.R2.L2** | **Sin.R3.L1** | **Sin.R3.L2** | **Sin.R4.L1** | **Sin.R4.L2** | **Sin.R5.L1** | **Sin.R5.L2** | **Sin.R6.L1** | **Sa.R1.L1** | **Sa.R1.L2** | **Sa.R1.L2.5** | **Sa.R1.L3** | **Sa.R2.L1** | **Sa.R2.L2** | **Sa.R3.L1** | **Sa.R3.L2** | **Sa.R4.L1** | **Sa.R4.L2** | **Tal.R1.L1** | **Tal.R1.L2** | **Tal.R2.L1** | **Tal.R3.L1** | **Tal.R4.L1** | **Tal.R4.L2** | **Mar.R1.L1** | **Mar.R1.L2** | **Mar.R2.L1** | **Mar.R2.L2** |
| --- | --- | --- | --- | --- | --- | --- | --- | --- | --- | --- | --- | --- | --- | --- | --- | --- | --- | --- | --- | --- | --- | --- | --- | --- | --- | --- | --- | --- | --- | --- | --- | --- |
| Baetidae | Nigrobaetis_sp. |  |  | 2 |  | 1 |  | 13 |  | 1 |  |  | 2 |  |  |  | 8 |  | 2 |  | 1 |  |  |  |  |  | 6 |  | 1 | 1 | 31 |  |
|  | Gen.New.A_sp1 |  | 1 |  | 9 |  |  |  |  |  |  |  | 1 | 1 |  |  | 6 |  | 6 |  |  | 5 |  |  |  |  |  |  |  |  |  | 1 |
|  | Liebebiella_sp. nov. |  |  | 1 |  | 37 |  |  |  | 6 |  |  |  |  |  |  |  |  |  |  |  |  |  |  | 1 |  | 1 |  |  |  | 5 |  |
|  | Gen.New.B_sp1 |  |  |  |  | 23 |  | 2 |  | 6 |  |  |  |  |  |  |  |  |  |  | 26 |  |  |  |  | 1 |  | 7 |  |  |  |  |
|  | Gen.New.B_sp2 |  |  |  |  | 5 |  | 1 |  |  |  |  |  |  |  |  |  |  |  |  | 30 |  |  |  | 46 |  |  |  | 1 |  |  |  |
|  | Labiobaetis_sp1 |  |  | 2 |  | 12 |  | 2 |  | 6 | 1 |  |  |  |  |  |  |  |  |  |  | 1 |  |  |  |  |  |  | 1 |  |  |  |
|  | Labiobaetis_sp2 | 3 | 9 | 3 | 1 | 3 | 6 |  |  |  |  |  |  |  |  | 6 |  |  |  |  |  | 2 |  | 9 |  |  |  | 3 | 3 |  |  | 37 |
|  | Baetis_sabahensis |  |  |  | 6 | 15 |  | 5 |  | 6 | 3 |  |  |  |  |  | 28 | 3 | 9 | 13 |  | 26 |  |  |  |  | 22 | 6 |  | 31 |  |  |
|  | Baetis_cf_sabahensis |  |  |  |  |  |  |  |  | 3 |  |  |  |  | 1 |  |  |  |  |  |  | 9 |  |  |  |  |  |  |  | 2 |  |  |
|  | Liebebiella_cf_vera |  |  | 7 |  | 42 |  | 10 |  | 42 | 19 |  |  | 6 |  | 1 |  |  | 1 | 1 | 204 | 2 | 15 |  | 89 | 7 | 3 | 20 | 11 | 1 | 22 |  |
|  | Bungona_sp1 |  | 2 |  | 14 |  |  | 2 | 1 | 13 |  | 1 | 1 |  |  |  |  |  | 2 | 3 |  |  |  | 2 | 36 |  | 8 | 89 |  | 6 | 3 |  |
|  | Bungona_sp2 |  | 1 |  |  |  |  | 1 |  |  |  |  |  |  |  |  |  |  |  | 1 |  |  |  |  |  |  |  |  |  |  |  |  |
|  | Procloeon_sp1 |  |  |  |  |  |  |  | 3 |  |  | 4 | 1 |  |  |  |  |  |  |  |  |  |  |  |  |  | 4 | 2 |  |  |  |  |
|  | Procloeon_sp2 |  |  |  |  |  |  |  | 4 |  |  |  |  |  |  |  |  |  |  |  |  | 1 |  |  |  |  |  | 51 |  | 3 |  |  |
|  | Gen.New.C_sp1 |  |  |  |  |  |  |  |  |  |  |  |  |  |  |  |  |  |  |  |  |  |  |  |  |  | 1 |  |  |  | 1 |  |
|  | Xyroasio_sp. |  |  |  |  |  |  |  |  |  |  |  |  |  |  |  |  |  |  |  |  |  |  |  |  |  |  | 14 |  |  |  |  |
|  | Labiobeatis_sp3 |  | 3 |  | 1 | 1 |  |  |  |  |  |  |  |  |  |  |  |  |  |  |  |  |  |  |  |  |  |  |  |  |  |  |
| Heptageniidae | Thalerosphyrussinuosus_sp. | |  |  | 3 |  | 1 | 38 |  | 2 | 2 | 45 |  | 11 | 17 |  | 1 | 11 | 45 | 122 |  |  |  |  |  |  |  | 122 |  | 63 |  |  |
|  | Thalerosphyruslamuriensis_sp. | 6 |  | 3 |  | 20 | 4 | 5 |  | 4 |  |  | 7 |  |  |  | 21 | 20 | 5 | 2 |  | 14 |  |  | 1 |  |  |  |  |  |  |  |
|  | Halerosphyrus_sp. |  |  |  |  |  |  | 4 |  |  |  |  |  |  |  |  |  |  |  |  |  |  |  |  | 1 |  | 7 |  |  |  |  |  |
|  | Rhithrogena_sp. |  |  |  |  |  |  |  |  |  |  |  |  |  |  |  |  |  |  |  |  |  |  |  | 9 |  | 1 |  |  |  | 6 |  |
|  | Asionurus_ulmeri | 40 | 20 |  | 50 |  | 2 |  | 5 |  |  |  |  |  |  | 16 |  | 9 |  | 1 |  |  |  | 5 |  |  |  | 1 |  |  |  | 24 |
|  | Compsoneuriella_sp. |  |  |  |  |  |  |  |  |  |  |  |  |  |  |  |  |  |  |  |  |  |  |  | 1 |  |  |  |  |  |  |  |
|  | Caenis_sp. | 1 |  |  | 5 | 1 |  | 1 |  |  |  |  | 1 | 2 |  | 1 |  |  | 3 | 7 | 12 | 4 | 1 |  | 4 |  | 3 | 1 | 14 | 4 | 1 | 1 |
|  | Drunella_sp. |  |  |  |  |  |  |  |  |  | 2 |  |  |  |  |  |  |  |  |  |  |  |  |  |  |  | 1 |  |  |  |  |  |
| Teloganodidae | Teloganopsis_media |  |  |  |  |  |  |  |  |  |  |  |  |  |  |  |  |  |  |  |  |  |  |  |  |  | 1 |  |  |  |  |  |
| Leptophlebiidae | Isca_sp. |  | 11 |  | 1 |  | 7 |  | 3 |  | 1 |  | 1 |  |  |  | 1 | 4 |  |  |  |  |  | 3 |  |  |  |  |  | 5 |  |  |
|  | Traulus_sp. |  |  |  |  |  |  |  | 1 |  |  |  |  |  |  |  |  |  |  |  |  |  |  |  |  |  |  |  |  |  |  |  |
|  | Euthraulus_sp. |  |  |  |  |  |  |  |  |  |  |  | 1 |  |  |  |  |  |  |  | 1 |  |  |  |  |  | 6 |  |  |  |  |  |
|  | Choroterpes_sp. |  |  |  |  |  |  |  |  |  |  |  |  |  |  |  |  | 1 |  |  |  |  |  |  |  |  |  |  |  |  |  |  |
| Teloganodidae | Dudgeodes_ulmeri |  |  |  | 1 |  | 1 |  | 18 |  |  | 2 |  | 4 |  |  | 1 | 1 | 1 |  |  | 5 | 1 |  |  | 1 | 2 | 7 |  |  |  | 12 |
| Tricorythidae | Sparsorythus_sp. |  |  |  |  |  |  |  |  |  |  |  |  |  |  |  |  |  |  |  |  |  | 19 |  |  | 5 | 3 |  |  |  | 2 |  |
| Prosopistomatidae | Prosopistoma_wouterae |  |  |  |  |  |  |  |  |  |  |  |  |  |  |  |  |  |  |  |  |  | 1 |  |  |  |  |  |  |  |  |  |
| Neoephemeridae | Potamanthellus_caenoides |  |  |  |  |  |  |  |  |  |  |  |  |  |  |  |  |  |  |  |  |  |  |  | 1 |  |  |  |  |  |  |  |
| Isonychiidae | Isonychia_sp. |  |  |  |  |  |  |  |  |  |  |  |  |  |  |  |  |  |  |  |  |  | 2 |  |  |  |  |  |  |  |  |  |

**S2 Results of the permutational manova using the adonis function in the vegan package^82^** (see text). Effect of watersheds, volcanoes and elevation (as well as double interactions) on species composition. Matrices were based on the Bray-Curtis distance measure. Statistical significance was assessed through 10,000 permutations.

| **Spatial features** | **d.f.** | **Sums of squares** | **Means of squares** | **F model** | **R2** | **P value** |
| --- | --- | --- | --- | --- | --- | --- |
| Watersheds | 6 | 1.3632 | 0.22720 | 1.0709 | 0.18351 | 0.4076 |
| Volcanoes | 3 | 0.9407 | 0.31355 | 1.4779 | 0.12663 | 0.1134 |
| Elevation | 1 | 1.1565 | 1.15651 | 5.4512 | 0.15569 | 0.0001* |
| Watersheds: Elevation | 6 | 1.2311 | 0.20518 | 0.9671 | 0.16573 | 0.5476 |
| Volcanoes: Elevation | 3 | 0.4031 | 0.13437 | 0.6334 | 0.05427 | 0.8705 |
| Residuals | 11 | 2.3338 | 0.21216 |  | 0.31417 |  |
| Total | 30 | 7.4283 |  |  | 1.00000 |  |

**S3 Mantel correlogram for the community level.** The correlogram was computed between a Bray-Curtis dissimilarity matrix of species presence/absence and a Euclidean matrix of pairwise elevation difference among communities. Studies sites are divided in different distance classes and are plotted according to their similarities and dissimilarities in terms of species composition. Only plain dotes are statistically significant. The correlogram was assessed through 10,000 permutations.

**S4 Fitted binary elevation vectors onto non-metric multidimensional scaling (NMDS) ordination of species abundance matrix using Bray-Curtis similarity index.** Elevation was split each 50 meters into binary vectors with “0” encoding for communities sampled below a given threshold and “1” for communities sampled above or exactly at the threshold. Using the Vegan package^82^, we performed a non-metric multidimensional scaling (NMDS) of species abundance matrix. By fitting all binary vectors onto the ordination, we retrieved R2 and considered the threshold with the highest R2 score (in bold) as the most meaningful cut-off between lowland and highland.

| **Elevation class** | **NMDS1** | **NMDS2** | **R2** | **P value** |
| --- | --- | --- | --- | --- |
| 500m | -0.82031 | -0.57192 | 0.2212 | 0.031 |
| 550m | -0.82031 | -0.57192 | 0.2212 | 0.031 |
| 600m | -0.93143 | -0.36391 | 0.4128 | 0.001 |
| 650m | -0.93143 | -0.36391 | 0.4128 | 0.001 |
| 700m | -0.93143 | -0.36391 | 0.4128 | 0.001 |
| 750m | -0.93143 | -0.36391 | 0.4128 | 0.001 |
| 800m | -0.93143 | -0.36391 | 0.4128 | 0.001 |
| **850m** | **-0.93403** | **-0.3572** | **0.4797** | **0.001** |
| 900m | -0.98 | -0.199 | 0.4341 | 0.002 |
| 950m | -0.98 | -0.199 | 0.4341 | 0.002 |
| 1000m | -0.92082 | -0.38998 | 0.415 | 0.004 |
| 1050m | -0.92227 | -0.38655 | 0.4053 | 0.003 |
| 1100m | -0.88538 | -0.46487 | 0.4544 | 0.002 |
| 1150m | -0.88538 | -0.46487 | 0.4544 | 0.002 |
| 1200m | -0.83669 | -0.54768 | 0.4676 | 0.001 |
| 1250m | -0.73049 | -0.68293 | 0.434 | 0.002 |
| 1300m | -0.64601 | -0.76333 | 0.4638 | 0.001 |
| 1350m | -0.61307 | -0.79003 | 0.3853 | 0.002 |
| 1400m | -0.61307 | -0.79003 | 0.3853 | 0.002 |
| 1450m | -0.63671 | -0.7711 | 0.3015 | 0.007 |
| 1500m | -0.63671 | -0.7711 | 0.3015 | 0.007 |
| 1550m | -0.63671 | -0.7711 | 0.3015 | 0.007 |
| 1600m | -0.63671 | -0.7711 | 0.3015 | 0.007 |
| 1650m | -0.7605 | -0.64933 | 0.2314 | 0.014 |

**S5 Similarity matrix of species composition between all sampling sites.** Matrix is composed of the number of shared species between sampling sites (upper side of matrix), the total number of species per site (bold numbers in diagonal), and the dissimilarity between sites based on Bray-Curtis index. Highland communities (above 850m) are in bold.

|  | **Sin.R1.L1** | **Sin.R1.L2** | **Sin.R2.L1** | **Sin.R2.L2** | **Sin.R3.L1** | **Sin.R3.L2** | Sin.R4.L1 | **Sin.R4.L2** | Sin.R5.L1 | **Sin.R5.L2** | Sin.R6.L1 | **Sa.R1.L1** | **Sa.R1.L2** | **Sa.R1.L2.5** | **Sa.R1.L3** | **Sa.R2.L1** | **Sa.R2.L2** | **Sa.R3.L1** | **Sa.R3.L2** | Sa.R4.L1 | **Sa.R4.L2** | Tal.R1.L1 | **Tal.R1.L2** | Tal.R2.L1 | Tal.R3.L1 | Tal.R4.L1 | **Tal.R4.L2** | **Mar.R1.L1** | **Mar.R1.L2** | Mar.R2.L1 | **Mar.R2.L2** |
| --- | --- | --- | --- | --- | --- | --- | --- | --- | --- | --- | --- | --- | --- | --- | --- | --- | --- | --- | --- | --- | --- | --- | --- | --- | --- | --- | --- | --- | --- | --- | --- |
| **Sin.R1.L1** | **4** | 2 | 2 | 3 | 3 | 3 | 2 | 1 | 1 | 0 | 0 | 2 | 1 | 0 | 3 | 1 | 2 | 2 | 3 | 1 | 3 | 1 | 2 | 2 | 0 | 1 | 3 | 2 | 1 | 1 | 3 |
| **Sin.R1.L2** | 0.64 | **7** | 1 | 6 | 2 | 3 | 2 | 3 | 1 | 1 | 1 | 3 | 1 | 0 | 2 | 2 | 2 | 2 | 3 | 0 | 2 | 0 | 4 | 1 | 0 | 1 | 3 | 1 | 2 | 1 | 3 |
| **Sin.R2.L1** | 0.60 | 0.85 | **6** | 1 | 6 | 2 | 4 | 0 | 5 | 2 | 0 | 2 | 1 | 0 | 2 | 2 | 1 | 3 | 2 | 2 | 4 | 1 | 1 | 3 | 1 | 3 | 2 | 4 | 2 | 3 | 1 |
| **Sin.R2.L2** | 0.57 | 0.29 | 0.88 | **10** | 4 | 5 | 4 | 4 | 3 | 3 | 3 | 4 | 4 | 1 | 3 | 5 | 5 | 6 | 5 | 1 | 5 | 2 | 4 | 2 | 1 | 4 | 7 | 2 | 5 | 2 | 5 |
| **Sin.R3.L1** | 0.60 | 0.78 | 0.29 | 0.62 | **11** | 2 | 8 | 0 | 7 | 3 | 0 | 3 | 2 | 0 | 3 | 3 | 2 | 5 | 4 | 5 | 6 | 2 | 1 | 5 | 2 | 5 | 5 | 6 | 4 | 4 | 2 |
| **Sin.R3.L2** | 0.40 | 0.54 | 0.67 | 0.38 | 0.76 | **6** | 2 | 3 | 2 | 2 | 2 | 2 | 2 | 1 | 2 | 4 | 5 | 3 | 3 | 0 | 3 | 1 | 3 | 1 | 1 | 1 | 4 | 1 | 2 | 0 | 3 |
| Sin.R4.L1 | 0.75 | 0.79 | 0.56 | 0.64 | 0.30 | 0.78 | **12** | 1 | 8 | 4 | 2 | 4 | 3 | 1 | 2 | 4 | 3 | 7 | 7 | 5 | 5 | 2 | 1 | 6 | 2 | 6 | 6 | 5 | 6 | 4 | 1 |
| **Sin.R4.L2** | 0.82 | 0.57 | 1.00 | 0.53 | 1.00 | 0.54 | 0.89 | **7** | 1 | 1 | 3 | 3 | 1 | 0 | 1 | 2 | 3 | 2 | 2 | 0 | 2 | 1 | 3 | 1 | 1 | 3 | 5 | 0 | 3 | 1 | 2 |
| Sin.R5.L1 | 0.86 | 0.88 | 0.38 | 0.70 | 0.33 | 0.75 | 0.27 | 0.89 | **10** | 4 | 2 | 3 | 2 | 2 | 1 | 4 | 3 | 6 | 5 | 3 | 5 | 1 | 1 | 4 | 2 | 5 | 5 | 3 | 6 | 4 | 0 |
| **Sin.R5.L2** | 1.00 | 0.85 | 0.67 | 0.63 | 0.65 | 0.67 | 0.56 | 0.86 | 0.50 | **6** | 1 | 1 | 2 | 1 | 1 | 3 | 3 | 3 | 3 | 1 | 3 | 1 | 1 | 1 | 1 | 3 | 3 | 2 | 4 | 1 | 0 |
| Sin.R6.L1 | 1.00 | 0.82 | 1.00 | 0.57 | 1.00 | 0.60 | 0.75 | 0.50 | 0.71 | 0.80 | **4** | 2 | 2 | 1 | 0 | 2 | 2 | 3 | 2 | 0 | 1 | 1 | 1 | 1 | 1 | 3 | 4 | 0 | 2 | 1 | 1 |
| **Sa.R1.L1** | 0.67 | 0.60 | 0.71 | 0.56 | 0.68 | 0.71 | 0.60 | 0.63 | 0.67 | 0.86 | 0.67 | **8** | 2 | 0 | 1 | 4 | 2 | 5 | 3 | 3 | 3 | 1 | 2 | 3 | 0 | 5 | 3 | 2 | 4 | 3 | 2 |
| **Sa.R1.L2** | 0.78 | 0.83 | 0.82 | 0.47 | 0.75 | 0.64 | 0.65 | 0.85 | 0.73 | 0.64 | 0.56 | 0.69 | **5** | 1 | 2 | 3 | 2 | 5 | 3 | 2 | 4 | 3 | 0 | 2 | 2 | 3 | 4 | 2 | 3 | 2 | 3 |
| **Sa.R1.L2.5** | 1.00 | 1.00 | 1.00 | 0.83 | 1.00 | 0.75 | 0.86 | 1.00 | 0.67 | 0.75 | 0.67 | 1.00 | 0.71 | **2** | 0 | 1 | 1 | 1 | 1 | 0 | 1 | 0 | 0 | 0 | 0 | 0 | 1 | 0 | 2 | 0 | 0 |
| **Sa.R1.L3** | 0.25 | 0.64 | 0.60 | 0.57 | 0.60 | 0.60 | 0.75 | 0.83 | 0.86 | 0.80 | 1.00 | 0.83 | 0.56 | 1.00 | **4** | 0 | 1 | 2 | 3 | 2 | 3 | 2 | 2 | 2 | 1 | 2 | 4 | 3 | 2 | 2 | 3 |
| **Sa.R2.L1** | 0.82 | 0.71 | 0.69 | 0.41 | 0.67 | 0.38 | 0.58 | 0.73 | 0.53 | 0.54 | 0.64 | 0.47 | 0.50 | 0.78 | 1.00 | **7** | 5 | 6 | 3 | 1 | 4 | 1 | 1 | 1 | 1 | 3 | 3 | 1 | 4 | 1 | 2 |
| **Sa.R2.L2** | 0.64 | 0.71 | 0.85 | 0.41 | 0.78 | 0.23 | 0.68 | 0.60 | 0.65 | 0.54 | 0.64 | 0.73 | 0.67 | 0.78 | 0.82 | 0.29 | **7** | 4 | 4 | 0 | 3 | 1 | 2 | 1 | 1 | 2 | 4 | 0 | 3 | 0 | 2 |
| **Sa.R3.L1** | 0.69 | 0.75 | 0.60 | 0.37 | 0.50 | 0.60 | 0.33 | 0.76 | 0.37 | 0.60 | 0.54 | 0.41 | 0.29 | 0.82 | 0.69 | 0.25 | 0.50 | **9** | 6 | 3 | 6 | 3 | 1 | 4 | 2 | 6 | 6 | 3 | 6 | 4 | 3 |
| **Sa.R3.L2** | 0.50 | 0.60 | 0.71 | 0.44 | 0.58 | 0.57 | 0.30 | 0.75 | 0.44 | 0.57 | 0.67 | 0.63 | 0.54 | 0.80 | 0.50 | 0.60 | 0.47 | 0.29 | **8** | 2 | 4 | 2 | 2 | 4 | 1 | 4 | 6 | 2 | 5 | 3 | 2 |
| Sa.R4.L1 | 0.80 | 1.00 | 0.67 | 0.88 | 0.41 | 1.00 | 0.44 | 1.00 | 0.63 | 0.83 | 1.00 | 0.57 | 0.64 | 1.00 | 0.60 | 0.85 | 1.00 | 0.60 | 0.71 | **6** | 2 | 2 | 0 | 3 | 2 | 4 | 3 | 4 | 3 | 3 | 1 |
| **Sa.R4.L2** | 0.57 | 0.76 | 0.50 | 0.50 | 0.43 | 0.63 | 0.55 | 0.78 | 0.50 | 0.63 | 0.86 | 0.67 | 0.47 | 0.83 | 0.57 | 0.53 | 0.65 | 0.37 | 0.56 | 0.75 | **10** | 3 | 1 | 3 | 2 | 4 | 6 | 4 | 5 | 2 | 4 |
| Tal.R1.L1 | 0.80 | 1.00 | 0.83 | 0.75 | 0.76 | 0.83 | 0.78 | 0.85 | 0.87 | 0.83 | 0.80 | 0.86 | 0.45 | 1.00 | 0.60 | 0.85 | 0.85 | 0.60 | 0.71 | 0.66 | 0.62 | **6** | 0 | 2 | 3 | 4 | 3 | 2 | 2 | 3 | 2 |
| **Tal.R1.L2** | 0.50 | 0.27 | 0.80 | 0.43 | 0.87 | 0.40 | 0.88 | 0.50 | 0.86 | 0.80 | 0.75 | 0.67 | 1.00 | 1.00 | 0.50 | 0.82 | 0.64 | 0.85 | 0.67 | 1.00 | 0.86 | 1.00 | **4** | 1 | 0 | 1 | 3 | 1 | 2 | 1 | 2 |
| Tal.R2.L1 | 0.71 | 0.88 | 0.62 | 0.80 | 0.52 | 0.87 | 0.44 | 0.88 | 0.60 | 0.87 | 0.86 | 0.67 | 0.73 | 1.00 | 0.71 | 0.88 | 0.88 | 0.57 | 0.56 | 0.62 | 0.70 | 0.75 | 0.86 | **10** | 1 | 6 | 3 | 3 | 3 | 5 | 1 |
| Tal.R3.L1 | 1.00 | 1.00 | 0.80 | 0.86 | 0.73 | 0.80 | 0.75 | 0.83 | 0.71 | 0.80 | 0.75 | 1.00 | 0.56 | 1.00 | 0.75 | 0.82 | 0.82 | 0.69 | 0.83 | 0.60 | 0.71 | 0.45 | 1.00 | 0.87 | **4** | 3 | 3 | 1 | 1 | 2 | 1 |
| Tal.R4.L1 | 0.89 | 0.91 | 0.71 | 0.68 | 0.62 | 0.90 | 0.56 | 0.74 | 0.60 | 0.71 | 0.68 | 0.57 | 0.70 | 1.00 | 0.79 | 0.73 | 0.82 | 0.50 | 0.65 | 0.62 | 0.68 | 0.64 | 0.89 | 0.62 | 0.68 | **15** | 6 | 3 | 5 | 8 | 2 |
| **Tal.R4.L2** | 0.63 | 0.68 | 0.78 | 0.36 | 0.57 | 0.56 | 0.50 | 0.50 | 0.55 | 0.67 | 0.50 | 0.70 | 0.53 | 0.86 | 0.50 | 0.68 | 0.58 | 0.43 | 0.40 | 0.67 | 0.45 | 0.68 | 0.63 | 0.74 | 0.63 | 0.56 | **12** | 3 | 6 | 3 | 4 |
| **Mar.R1.L1** | 0.60 | 0.85 | 0.33 | 0.75 | 0.29 | 0.83 | 0.44 | 1.00 | 0.63 | 0.67 | 1.00 | 0.71 | 0.64 | 1.00 | 0.40 | 0.85 | 1.00 | 0.60 | 0.71 | 0.33 | 0.50 | 0.69 | 0.80 | 0.65 | 0.80 | 0.71 | 0.67 | **6** | 3 | 3 | 2 |
| **Mar.R1.L2** | 0.85 | 0.75 | 0.73 | 0.47 | 0.60 | 0.73 | 0.43 | 0.65 | 0.37 | 0.47 | 0.69 | 0.53 | 0.57 | 0.64 | 0.69 | 0.50 | 0.63 | 0.33 | 0.41 | 0.60 | 0.47 | 0.75 | 0.69 | 0.70 | 0.85 | 0.58 | 0.43 | 0.60 | **9** | 4 | 1 |
| Mar.R2.L1 | 0.83 | 0.87 | 0.57 | 0.78 | 0.58 | 1.00 | 0.60 | 0.88 | 0.56 | 0.86 | 0.83 | 0.63 | 0.69 | 1.00 | 0.67 | 0.87 | 1.00 | 0.53 | 0.63 | 0.57 | 0.78 | 0.60 | 0.83 | 0.58 | 0.67 | 0.30 | 0.70 | 0.57 | 0.53 | **8** | 1 |
| **Mar.R2.L2** | 0.33 | 0.50 | 0.82 | 0.33 | 0.75 | 0.45 | 0.88 | 0.69 | 1.00 | 1.00 | 0.78 | 0.69 | 0.40 | 1.00 | 0.33 | 0.67 | 0.67 | 0.57 | 0.69 | 0.82 | 0.47 | 0.67 | 0.56 | 0.88 | 0.78 | 0.80 | 0.53 | 0.64 | 0.86 | 0.85 | **5** |

**S6 Spatial genetic structure plotted along the elevation gradient for four mayfly species.** Pie-charts represent the proportion of samples assigned to each cluster within each sampling location. Spatial structure was computed using STRUCTURE^87^ on restriction-site-associated-DNA sequencing (RADseq) data, by examining *K* values ranging from 1 to 8, and replicating analyses five times. The majority-rule criterion (>0.5 in the assignment probability) was applied to assign samples to a given cluster. The optimal number of genetic clusters was established by inspection of the likelihood function. Pie charts correspond to the sampling sites listed in table S1; x-axis exhibits the volcano range (Mar. = Marapi; Sag. = Sago; Sin. = Singgalang and Tal. = Talamau) and watershed of each sampling site and y-axis depicts elevation in meters. The horizontal red line represents the cut-off at 850m between lowland and highland populations.


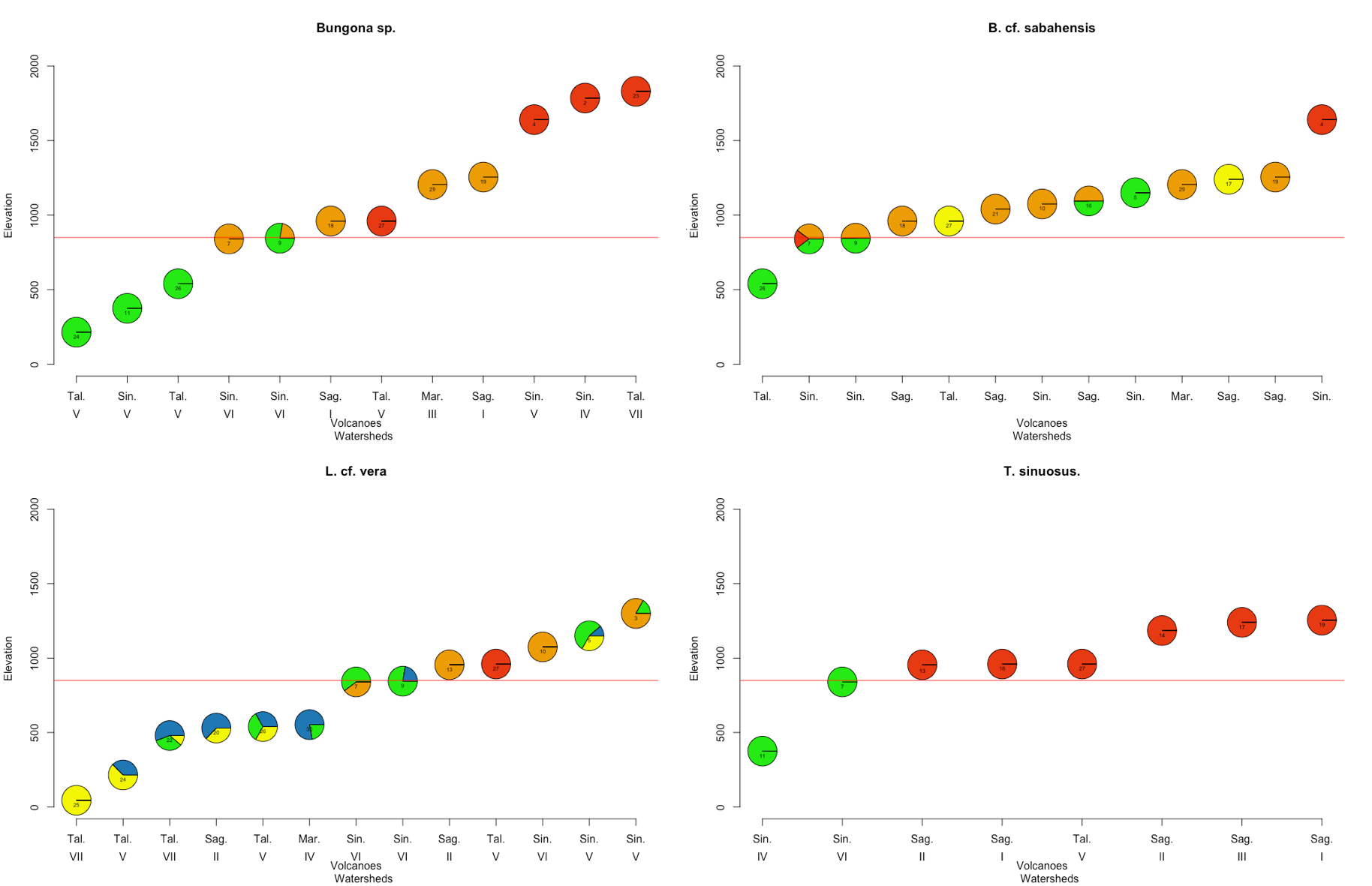


**S7 Analyses of molecular variance (AMOVA) for four Ephemeroptera species with wide ecological distributions, using restriction-site-associated-DNA sequencing (RADseq).** AMOVA were conducted using three different regional levels, (a) watersheds, (b) volcanoes, and (c) elevation—considered as a categorical trait (lowland/highland). The cutoff between lowland and highland was determined by the elevation that best predicted changes in community composition along the gradient (see Supplementary material S4). *P* value estimation was based on 1,000 permutations and corrected for multiple-test bias using Bonferroni correction (alpha = 0.05/3 = 0.0167). Fixation indexes are calculated as follows: Frt = estimated variance among region / total estimated variance (among populations + among individuals + among regions); Fsr = estimated variance among populations / total estimated variance; Fst = estimated variance (among region + among populations) / total estimated variance.

| **Source of variation** | **d.f.** | | **Variance components** | **Percentage variation** | **Fixation index** | **P value** |  |
| --- | --- | --- | --- | --- | --- | --- | --- |
| 1. ***Populations grouped according to major watersheds*** | | | | | | | |
| Among watersheds | | | | | | | |
| *Bungona sp.* | | 5 | 0.000 | 0% | Frt = -0.094 | 1.000 |  |
| *Liebebiella cf. vera* | | 4 | 0.000 | 0% | Frt = -0.062 | 1.000 |  |
| *B. c.f. sabahensis* | | 4 | 72.163 | 23% | Frt = 0.230 | 0.001 | * |
| *T. sinuosus* | | 5 | 50.075 | 38% | Frt = 0.378 | 0.001 | * |
| Among populations within region | | | | | | | |
| *Bungona sp.* | | 6 | 120.219 | 41% | Fsr = 0.410 | 0.001 | * |
| *Liebebiella cf. vera* | | 8 | 45.969 | 34% | Fsr = 0.342 | 0.001 | * |
| *B. c.f. sabahensis* | | 8 | 109.379 | 35% | Fsr = 0.454 | 0.001 | * |
| *T. sinuosus* | | 2 | 0.000 | 0% | Fsr = -0.001 | 0.401 |  |
| Among individuals within populations | | | | | | | |
| *Bungona sp.* | | 50 | 163.558 | 56% | Fst = 0.355 | 0.001 | * |
| *Liebebiella cf. vera* | | 78 | 86.492 | 64% | Fst = 0.302 | 0.001 | * |
| *B. c.f. sabahensis* | | 71 | 126.176 | 40% | Fst = 0.580 | 0.001 | * |
| *T. sinuosus* | | 54 | 77.397 | 58% | Fst = 0.378 | 0.001 | * |
| Total | | | | | | | |
| *Bungona sp.* | | 123 | 293.333 |  |  |  |  |
| *Liebebiella cf. vera* | | 181 | 134.246 |  |  |  |  |
| *B. c.f. sabahensis* | | 167 | 313.123 |  |  |  |  |
| *T. sinuosus* | | 123 | 132.569 |  |  |  |  |
| 1. ***Populations grouped according to volcano*** | | | | | | | |
| Among volcanoes | | | | | | | |
| *Bungona sp.* | | 3 | 0.000 | 0% | Frt = -0.011 | 0.860 |  |
| *Liebebiella cf. vera* | | 3 | 1.998 | 2% | Frt = 0.016 | 0.033 |  |
| *B. c.f. sabahensis* | | 3 | 57.610 | 18% | Frt = 0.183 | 0.001 | * |
| *T. sinuosus* | | 2 | 69.308 | 46% | Frt = 0.456 | 0.001 | * |
| Among populations within region | | | | | | | |
| *Bungona sp.* | | 8 | 104.435 | 38% | Fsr = 0.376 | 0.001 | * |
| *Liebebiella cf. vera* | | 9 | 38.002 | 30% | Fsr = 0.301 | 0.001 | * |
| *B. c.f. sabahensis* | | 9 | 126.306 | 40% | Fsr = 0.490 | 0.001 | * |
| *T. sinuosus* | | 5 | 0.151 | 0% | Fsr = 0.002 | 0.386 |  |
| Among individuals within populations | | | | | | | |
| *Bungona sp.* | | 50 | 163.558 | 59% | Fst = 0.369 | 0.001 | * |
| *Liebebiella cf. vera* | | 78 | 86.492 | 67% | Fst = 0.312 | 0.001 | * |
| *B. c.f. sabahensis* | | 71 | 126.176 | 40% | Fst = 0.583 | 0.001 | * |
| *T. sinuosus* | | 54 | 77.397 | 51% | Fst = 0.457 | 0.001 | * |
| Total | | | | | | | |
| *Bungona sp.* | | 123 | 277.549 |  |  |  |  |
| *Liebebiella cf. vera* | | 181 | 128.278 |  |  |  |  |
| *B. c.f. sabahensis* | | 167 | 315.496 |  |  |  |  |
| *T. sinuosus* | | 123 | 151.953 |  |  |  |  |
| 1. ***Populations grouped according to elevation*** | | | | | | | |
| Among elevation categories | |  |  |  |  |  |  |
| *Bungona sp.* | | 1 | 108.134 | 34% | Frt = 0.335 | 0.001 | * |
| *Liebebiella cf. vera* | | 1 | 19.209 | 14% | Frt = 0.140 | 0.001 | * |
| *B. c.f. sabahensis* | | 1 | 34.679 | 11% | Frt = 0.107 | 0.001 | * |
| *T. sinuosus* | | 1 | 77.794 | 47% | Frt = 0.474 | 0.001 | * |
| Among populations within region | | | | | | | |
| *Bungona sp.* | | 10 | 41.084 | 13% | Fsr = 0.192 | 0.001 | * |
| *Liebebiella cf. vera* | | 11 | 31.711 | 23% | Fsr = 0.269 | 0.001 | * |
| *B. c.f. sabahensis* | | 11 | 156.983 | 49% | Fsr = 0.544 | 0.001 | * |
| *T. sinuosus* | | 6 | 3.891 | 2% | Fsr = 0.045 | 0.011 | * |
| Among individuals within populations | | | | | | | |
| *Bungona sp.* | | 50 | 163.558 | 51% | Fst = 0.463 | 0.001 | * |
| *Liebebiella cf. vera* | | 78 | 84.539 | 62% | Fst = 0.371 | 0.001 | * |
| *B. c.f. sabahensis* | | 71 | 126.176 | 39% | Fst = 0.593 | 0.001 | * |
| *T. sinuosus* | | 54 | 77.397 | 47% | Fst = 0.498 | 0.001 | * |
| Total | | | | | | | |
| *Bungona sp.* | | 123 | 322.333 |  |  |  |  |
| *Liebebiella cf. vera* | | 181 | 137.780 |  |  |  |  |
| *B. c.f. sabahensis* | | 167 | 323.243 |  |  |  |  |
| *T. sinuosus* | | 123 | 164.179 |  |  |  |  |

**S8 Most discriminating cut-off between lowland and highland populations.** Calculations were performed for each species separately by comparing Frt values for each elevation class. For each species, the highest Frt value was retained as the altitudinal cut off between lowland and highland populations. In order to ensure at least two populations per elevation class, we selected the second highest Frt value in cases where the highest Frt value fell at one end of the elevation gradient. Elevation classes were calculated as the median elevation between two successive sampling stations.


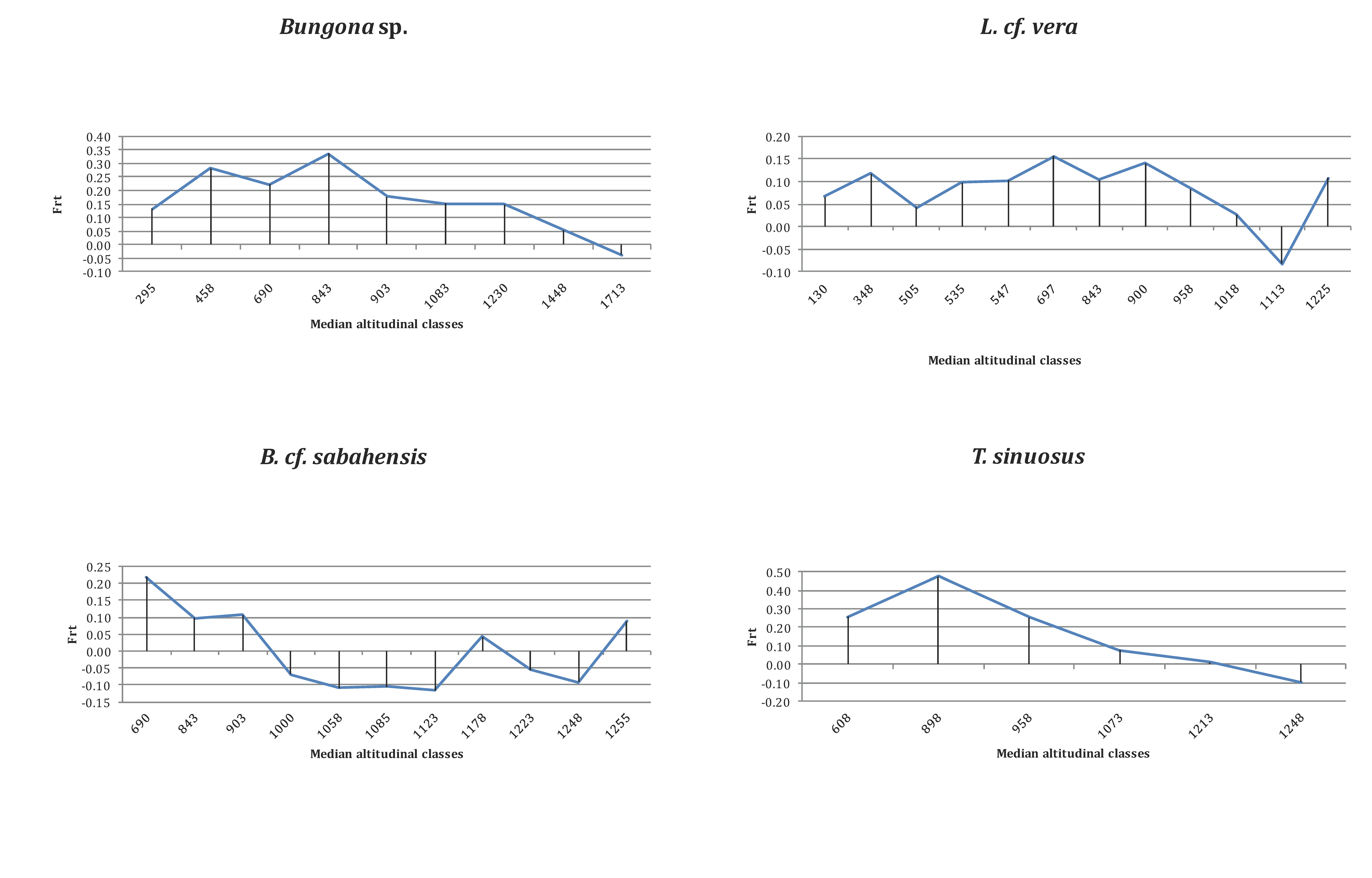


**TableS9 Results of the environmental factors fitted onto the non-metric multidimensional scaling (NMDS) based on the Bray-Curtis dissimilarity index of the species presence/absence matrix as well as the species genetic distance matrix.** Analyses were performed true 1000 permutations with the vegan package^82^. Because of the high number of missing values in the temperature analyses were performed without the factor to reduce biases in sample size.

| **Levels** | **Data points** | **Factors** | **NMDS1** | **NMDS2** | **R^2^** | **Pr(>r)** |
| --- | --- | --- | --- | --- | --- | --- |
| Communities | 23/31 | pH | 0.97971 | 0.20042 | 0.5156 | 0.003* |
|  |  | Velocity | 0.77973 | 0.62611 | 0.5902 | 0.001* |
| *Bungona sp.* | 56/62 | pH | 0.99584 | 0.09116 | 0.6452 | 0.001* |
|  |  | Velocity | 0.38077 | -0.92467 | 0.8097 | 0.001* |
| *L. cf vera* | 57/91 | pH | -0.97638 | -0.21605 | 0.3244 | 0.001* |
|  |  | Velocity | -0.79124 | -0.61151 | 0.1365 | 0.016* |
| *B. c.f. sabahensis* | 74/84 | pH | -0.37772 | -0.92592 | 0.5584 | 0.001* |
|  |  | Velocity | 0.01052 | -0.99994 | 0.3408 | 0.001* |
| *T.sinuosus* | 26/62 | pH | 0.95695 | -0.29026 | 0.0911 | 0.353 |
|  |  | Velocity | 0.37013 | -0.92898 | 0.0738 | 0.438 |

**Table**

**S10 Non-metric multidimensional scaling (NMDS) with stable solution from random starts based on the Bray–Curtis similarity index of the species presence/absence matrix.** To visualize clustering of species within communities and individuals within species along the elevational gradient and to determine how well the ordination fits the elevation variable, we fitted and plotted a generalized additive model (GAM) using a 2D smooth surface onto the NMDS site scores. The NMDS analyses were performed with the metaMDS function implemented in the vegan package^82^ and isoclines were fitted to the plot the ordisurf function. Furthermore, to visually inspect the accuracy of the model, we added a “spider” diagram onto the ordination plot connecting communities or individuals from highlands or lowland to their group centroid.

**S11 Time-calibrated trees.** Clock-constrained molecular phylogeny using an a priori substitution rate (3.68%) with a Bayesian relaxed clock for for **(a)** *L. cf vera* , **(b)** *B. cf. sabahensis*, and **(c)** *T. sinuosus* (results for *Bungona sp.* are shown as Figure 4 in the main text). The analyses were based on the concatenated intron data sets. Time axes are given in million years ago (mya). Grey bars indicate 95% highest posterior density (HPD) intervals. Black filled circles indicate strongly supported nodes (Bayesian posterior probability (BPP) ≥ 0.95) and white circles moderately supported nodes (BPP ≥ 0.90). Colors to the left of terminal labels indicate the watersheds, elevations, volcanos, and Structure clusters (Supplementary Information S6).

a)


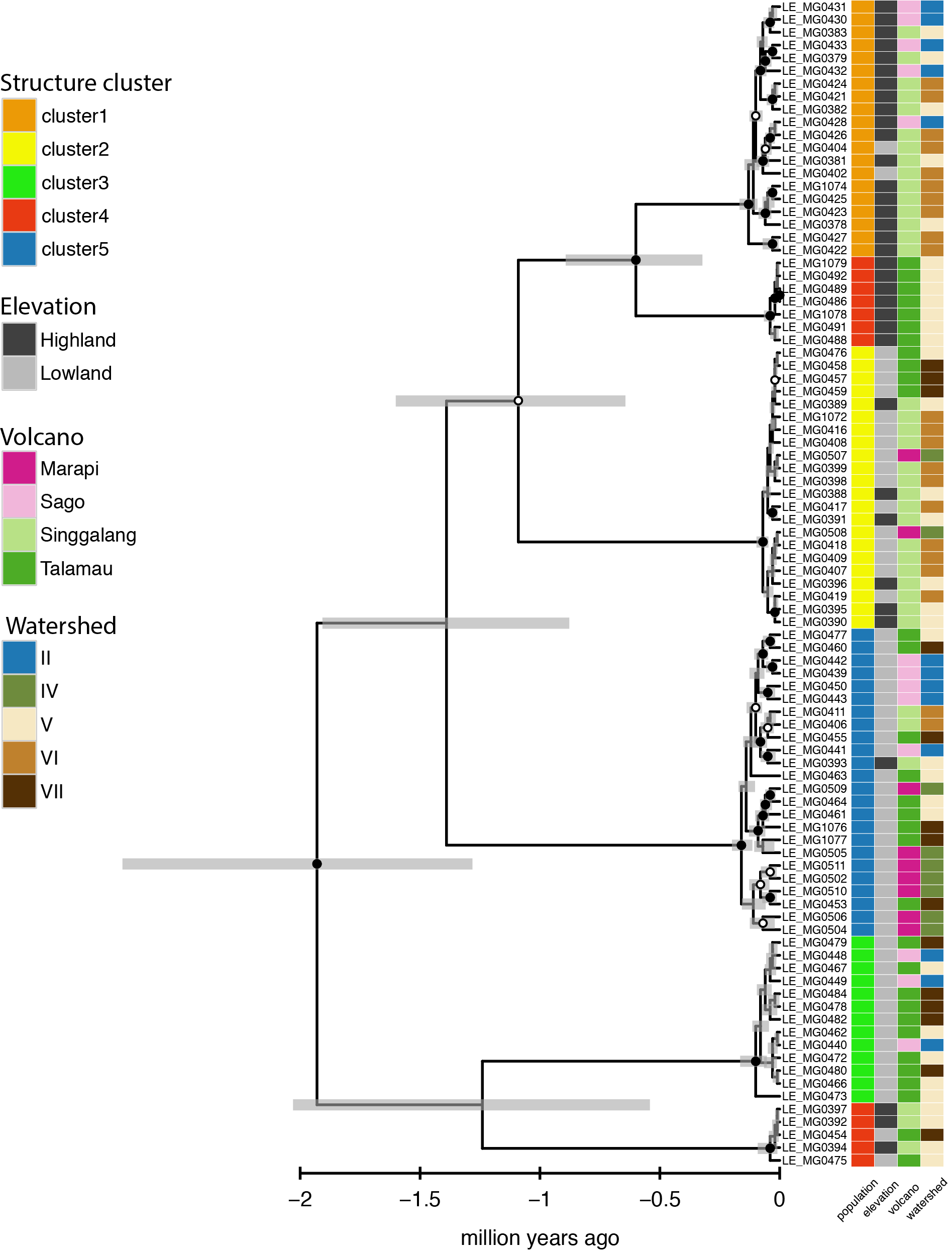


b)


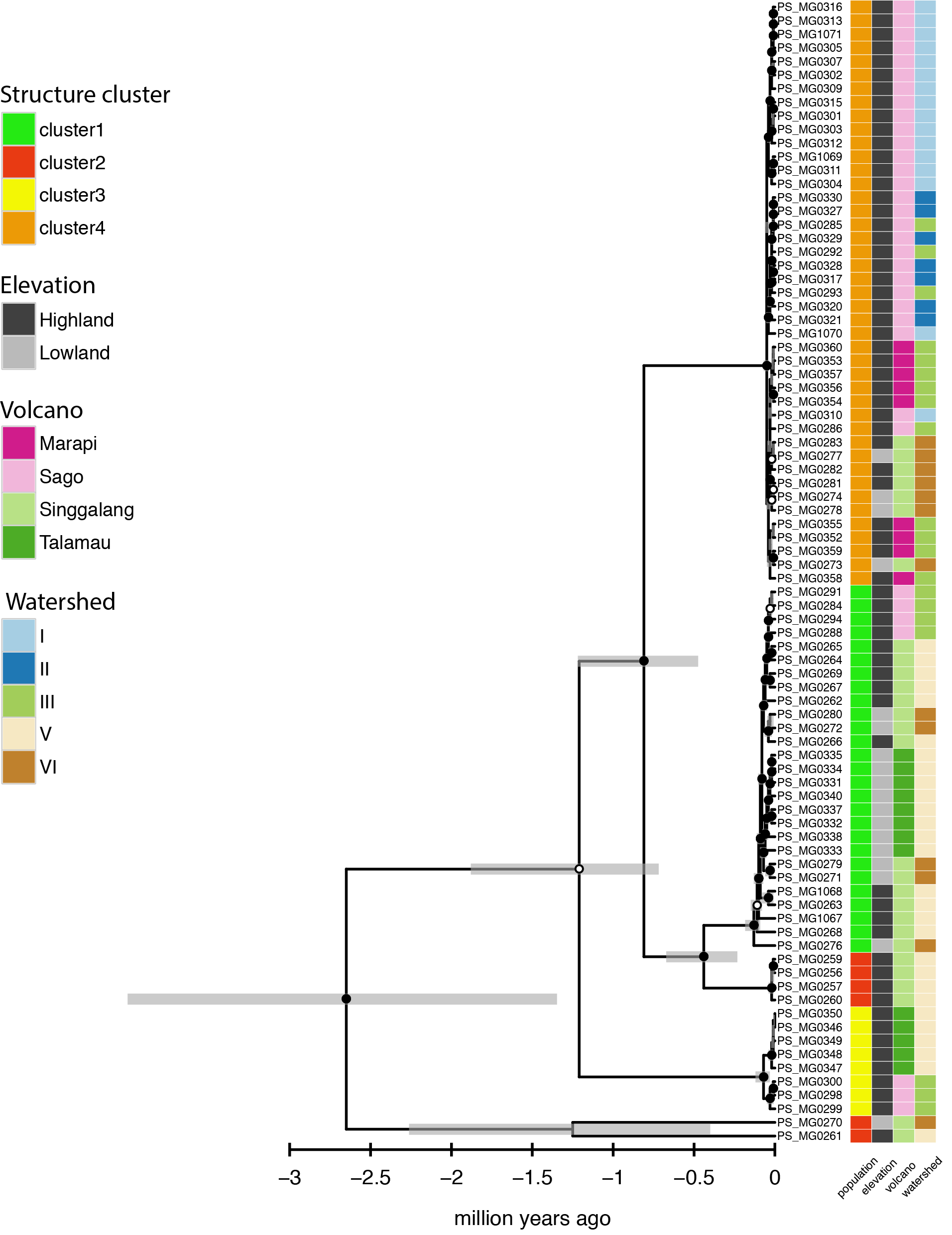


c)


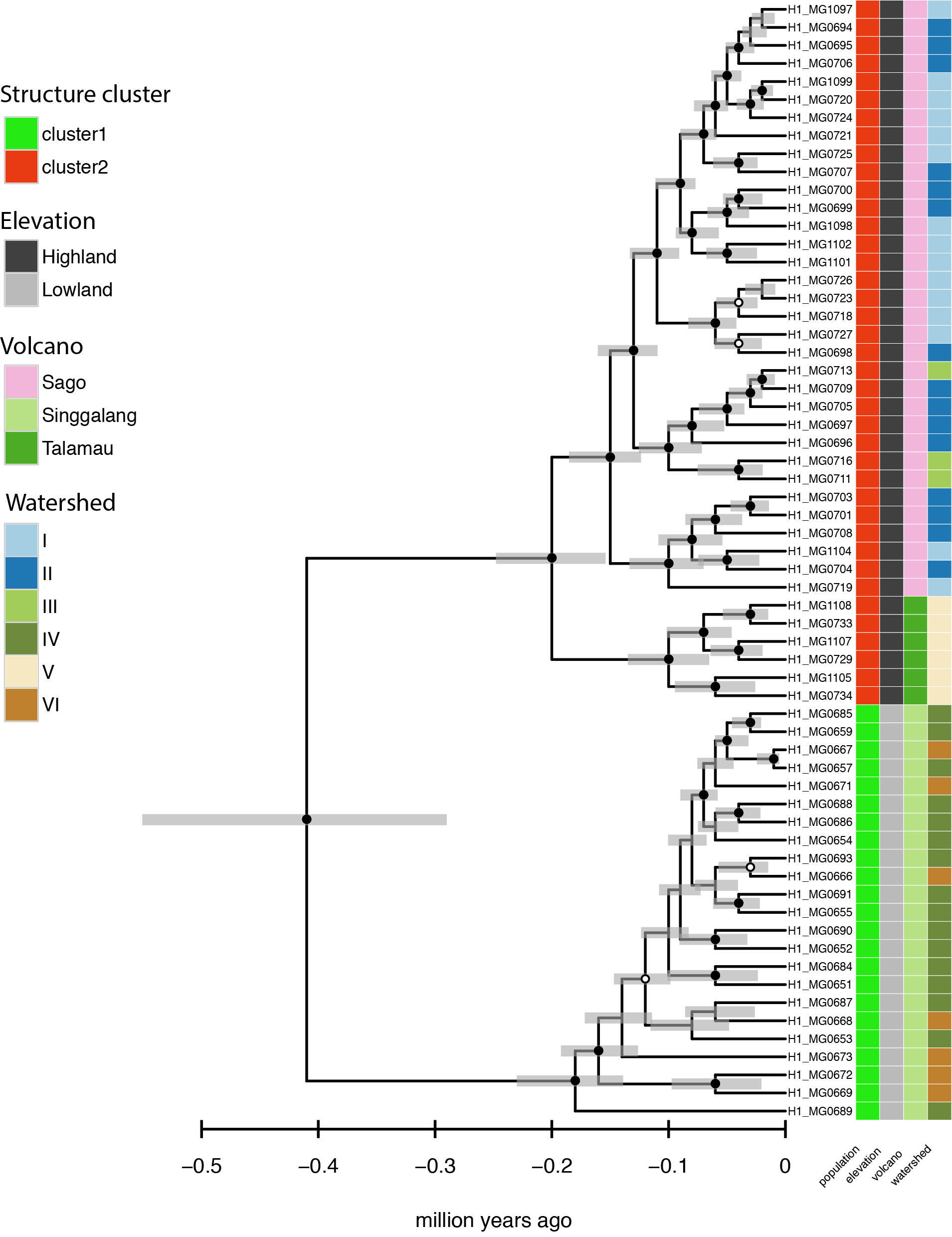


**S12 Coalescent-based trees.** Phylogenetic trees based on unlinked single nucleotide polymorphisms for **(a)** *Bungona sp.*, **(b)** *L. cf vera* , **(c)** *B. cf. sabahensis*, and **(d)** *T. sinuosus*. Filled circles on the nodes indicate the proportion of bootstrap replicates that support the respective relationhsip (black circles ≥ 0.95 ; and grey circles ≥ 0.75). Colors to the left of terminal labels indicate the clusters (Supplementary Information S6).

a)


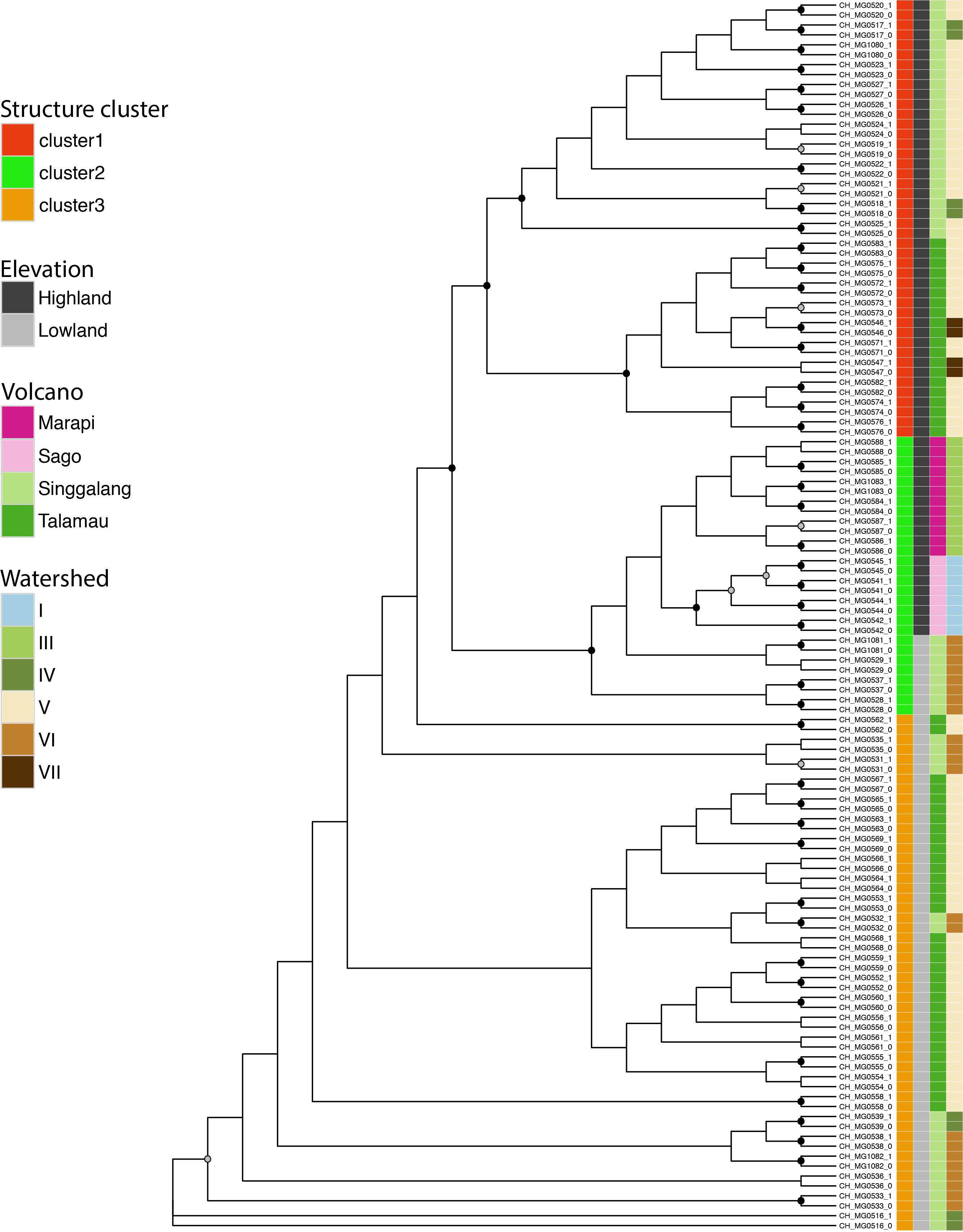


b)


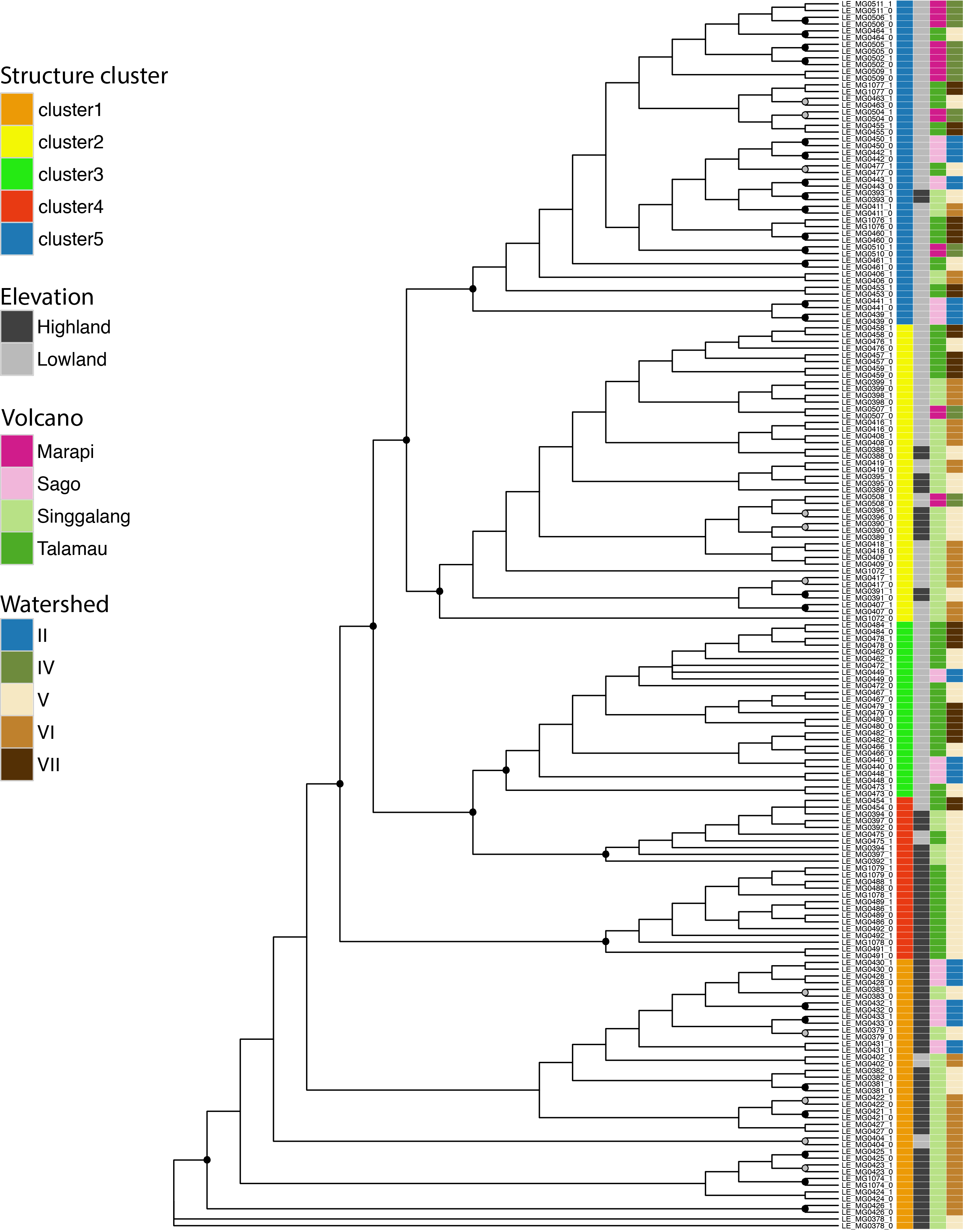


c)


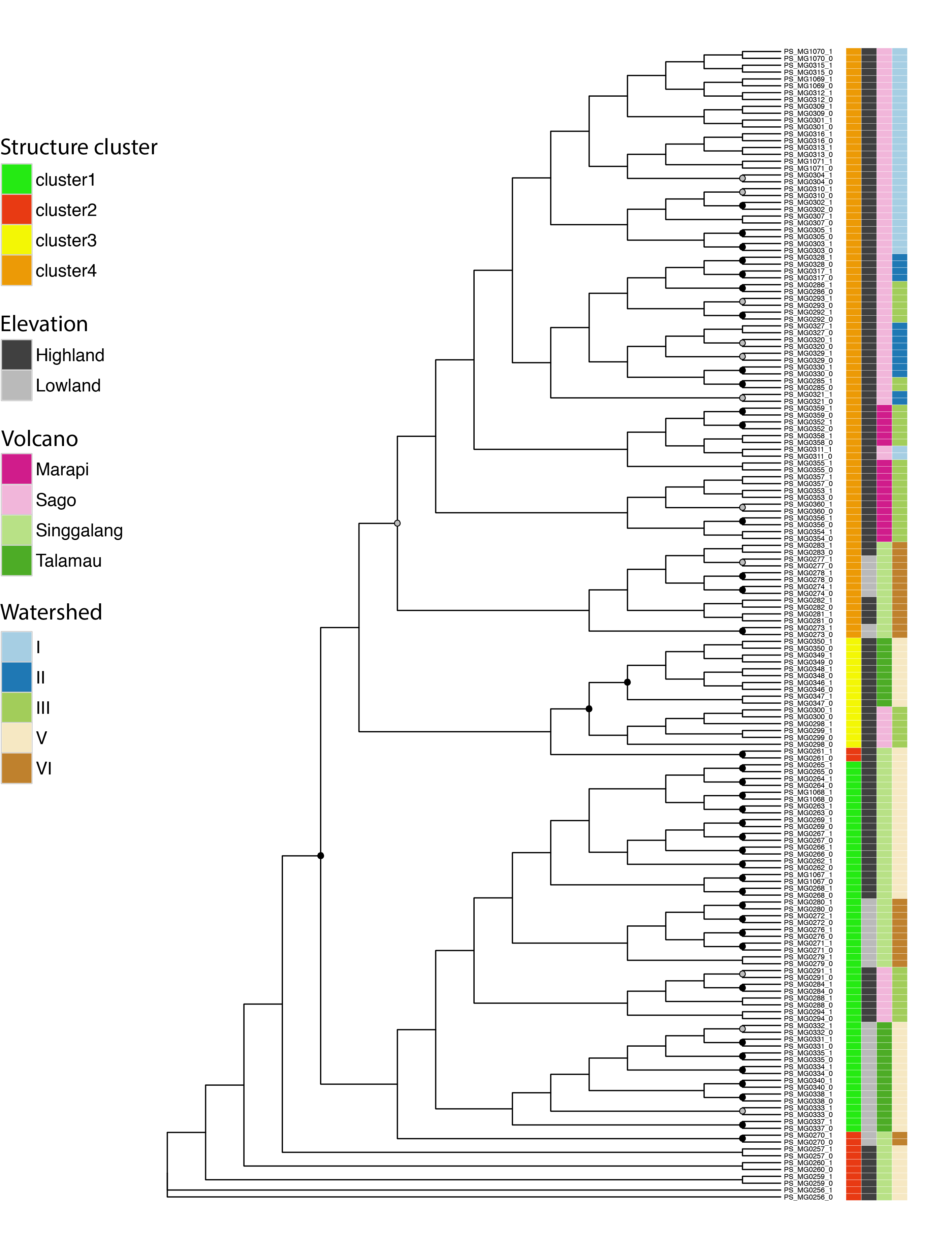


d)


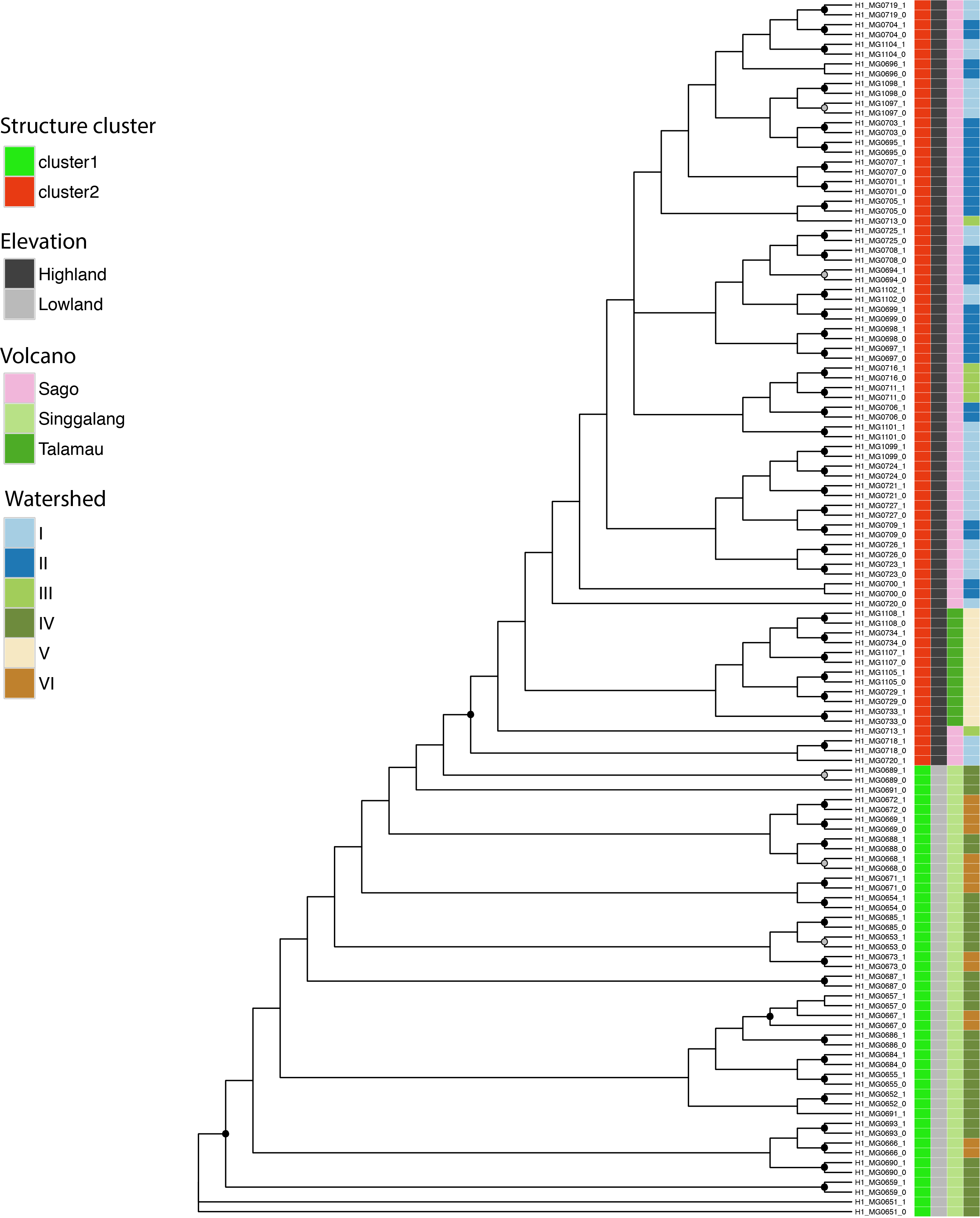


**S13 Plots of elevation against environmental parameters.** In each sampling sites, water temperature, pH and stream velocity where recorded and plotted against elevation in order to visually illustrate covariance between these variables. The slope of the corresponding linear regression is shown as a red line.


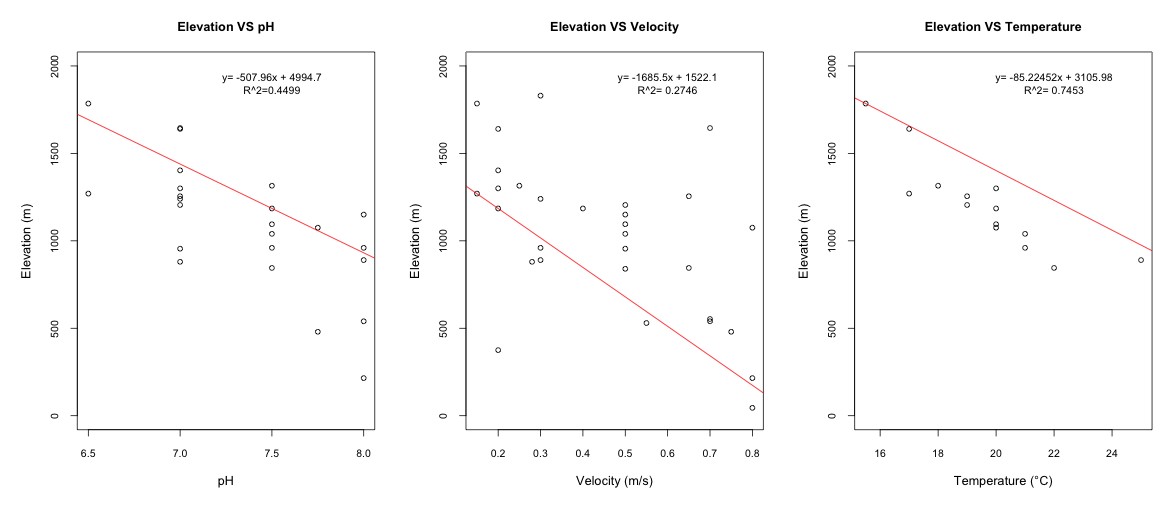


**S14 Distribution of the 31 sampled mayfly communities (black dots) in West Sumatra, Indonesia***.* The elevation model indicates low and high elevation areas along a colour continuum ranging from green to red. The four labelled volcanoes show up as sky islands on the elevation map. Bold broken lines represent drainage basin boundaries between the seven sampled watersheds (roman numerals). Maps were generated with the Quantum GIS geographic

information system (QGIS 2.18; QGIS Development Team, 2016. QGIS Geographic Information System. Open Source Geospatial Foundation Project; available at https://www.qgis.org).

**S15 Sampling locations and complementary information.** Please see Figure 1 for the distribution of the 31 communities and 7 watersheds (I to VII).

| **Sampling site** | **Volcano** | **River** | **Elevation** | **Elevation category using the 850 meters threshold** | **Watershed** | **Coord long.** | **Coord lat.** |
| --- | --- | --- | --- | --- | --- | --- | --- |
| 1 | Singgalang | Pagu Pagu | 1185m | Highland | IV | E100.37855 | S00.40332 |
| 2 | Singgalang | Pagu Pagu | 1785m | Highland | IV | E100.35757 | S00.39234 |
| 3 | Singgalang | Caruak | 1300m | Highland | V | E100.37823 | S00.38234 |
| 4 | Singgalang | Caruak | 1640m | Highland | V | E100.356799 | S00.384283 |
| 5 | Singgalang | Sianok | 1150m | Highland | V | E100.321875 | S00.33249 |
| 6 | Singgalang | Sianok | 1315m | Highland | V | E100.311826 | S00.311826 |
| 7 | Singgalang | Airjernih | 840m | Lowland | VI | E100.27888 | S00.40202 |
| 8 | Singgalang | Airjernih | 1270m | Highland | VI | E100.298846 | S00.407091 |
| 9 | Singgalang | Mangyih | 845m | Lowland | VI | E100.27619 | S00.39257 |
| 10 | Singgalang | Mangyih | 1075m | Highland | VI | E100.29420 | S00.38068 |
| 11 | Singgalang | Anai | 375m | Lowland | IV | E100.33424 | S00.48714 |
| 12 | Sago | Simbukan | 880m | Highland | II | E100.68707 | S00.28551 |
| 13 | Sago | Simbukan | 955m | Highland | II | E100.685088 | S00.289975 |
| 14 | Sago | Simbukan | 1185m | Highland | II | E100.683321 | S00.304872 |
| 15 | Sago | Simbukan | 1645m | Highland | II | E100.678873 | S00.315530 |
| 16 | Sago | Kobun | 1095m | Highland | III | E100.65913 | S00.37590 |
| 17 | Sago | Kobun | 1240m | Highland | III | E100.66244 | S00.36728 |
| 18 | Sago | Tampo | 960m | Highland | I | E100.69588 | S00.37221 |
| 19 | Sago | Tampo | 1255m | Highland | I | E100.68382 | S00.34365 |
| 20 | Sago | Kaligain | 530m | Lowland | II | E100.66368 | S00.24335 |
| 21 | Sago | Kaligain | 1040m | Highland | II | E100.66886 | S00.30028 |
| 22 | Talamau | Karumiang | 480m | Lowland | VII | E099.93419 | N00.12014 |
| 23 | Talamau | Karumiang | 1830m | Highland | VII | E099.96847 | N00.08920 |
| 24 | Talamau | Batimah | 215m | Lowland | V | E100.07745 | N00.01152 |
| 25 | Talamau | Matibo | 45m | Lowland | VII | E099.82219 | N00.07297 |
| 26 | Talamau | Pularian | 540m | Lowland | V | E100.0004 | N00.01653 |
| 27 | Talamau | Pularian | 960m | Highland | V | E099.99008 | N00.03757 |
| 28 | Marapi | NA1 | 890m | Highland | III | E100.554850 | S00.36620 |
| 29 | Marapi | NA1 | 1205m | Highland | III | E100.511708 | S00.359247 |
| 30 | Marapi | Anai | 553m | Lowland | IV | E100.36899 | S00.47481 |
| 31 | Marapi | NA2 | 1403m | Highland | IV | E100.424733 | S00.394237 |

**S16 – Restriction site-associated DNA sequencing (RADseq) protocol**

**Double digest Restriction site associated DNA (RAD) sequencing protocol**

This protocol is a modified version of A. Mastretta-Yanes (2014 *Restriction site-associated DNA sequencing, genotyping error estimation and de novo assembly optimization for population genetic inference)*

Summary of main modifications:

- Polymerase used: Q5 high fidelity polymerase instead of phusion Taq
- PCR cycles reduced to 15 (originally 20) in order to minimize PCR errors
- Size selection using Blue Pippin instead of agarose gel extraction
- Purification and equimolar pool of PCR products. This step is added to allow a more equal coverage of all samples during sequencing.

96 SbfI adapters were used. The barcodes were designed using the Python script at https://bioinf.eva.mpg.de/multiplex/

**Table 1.** Oligo sequences for PCR primers and MseI adaptors (5’-3’)

| **ID** | **Sequence** | | **Index sequence **** |
| --- | --- | --- | --- |
| ILLPCR2_ind01 | A*A*T GAT ACG GCG ACC ACC GAG ATC TAC ACT CTT TCC CTA CAC GAC GCT CTT CCG ATC T | | ATCACG |
| ILLPCR2_ind02 | C*A*A GCA GAA GAC GGC ATA CGA GAT CGT GAT GTG ACT GGA GTT CAG ACG TGT GC | | CGATGT |
| ILLPCR2_ind03 | C*A*A GCA GAA GAC GGC ATA CGA GAT ACA TCG GTG ACT GGA GTT CAG ACG TGT GC | | TTAGGC |
| ILLPCR2_ind04 | C*A*A GCA GAA GAC GGC ATA CGA GAT GCC TAA GTG ACT GGA GTT CAG ACG TGT GC | | TGACCA |
| ILLPCR2_ind05 | C*A*A GCA GAA GAC GGC ATA CGA GAT TGG TCA GTG ACT GGA GTT CAG ACG TGT GC | | ACAGTG |
| ILLPCR2_ind06 | C*A*A GCA GAA GAC GGC ATA CGA GAT CAC TGT GTG ACT GGA GTT CAG ACG TGT GC | | GCCAAT |
| ILLPCR2_ind07 | C*A*A GCA GAA GAC GGC ATA CGA GAT ATT GGC GTG ACT GGA GTT CAG ACG TGT GC | | CAGATC |
| ILLPCR2_ind08 | C*A*A GCA GAA GAC GGC ATA CGA GAT GAT CTG GTG ACT GGA GTT CAG ACG TGT GC | | ACTTGA |
| ILLPCR2_ind09 | C*A*A GCA GAA GAC GGC ATA CGA GAT TCA AGT GTG ACT GGA GTT CAG ACG TGT GC | | GATCAG |
| ILLPCR2_ind10 | C*A*A GCA GAA GAC GGC ATA CGA GAT CTG ATC GTG ACT GGA GTT CAG ACG TGT GC | | TAGCTT |
| ILLPCR2_ind11 | C*A*A GCA GAA GAC GGC ATA CGA GAT AAG CTA GTG ACT GGA GTT CAG ACG TGT GC | | GGCTAC |
| ILLPCR2_ind12 | C*A*A GCA GAA GAC GGC ATA CGA GAT GTA GCC GTG ACT GGA GTT CAG ACG TGT GC | | CTTGTA |
| ILLPCR1 | A*A*T GAT ACG GCG ACC ACC GAG ATC TAC ACT CTT TCC CTA CAC GAC GCT CTT CCG ATC T | |  |
| P2.1 MseI | GTG ACT GGA GTT CAG ACG TGT GCT CTT CCG ATC T |  | |
| P2.2 MseI | /5Phos/TAA GAT CGG AAG AGC GAG AAC AA |  | |

**Table 2.** Specific P1 oligo sequences for SbfI adapters (5’-3’). Note that P2, ILLPCR1 and ILLPCR2 oligos are as in Mastretta-Yanes et al. (2015).

| **code #** | **barcode seq** | **ID** | **P1.1 sequence** | **ID** |  | **P1.2 sequence** |
| --- | --- | --- | --- | --- | --- | --- |
| 1 | ACGG | P1_01.1 | ACACTCTTTCCCTACACGACGCTCTTCCGATCTACGGTGCA | P1_01.2 | CCGTAGATCGGAAGAGCGTCGTGTAGGGAAAGAGTGT | CCGTAGATCGGAAGAGCGTCGTGTAGGGAAAGAGTGT |
| 2 | TGCT | P1_02.1 | ACACTCTTTCCCTACACGACGCTCTTCCGATCTTGCTTGCA | P1_02.2 | AGCAAGATCGGAAGAGCGTCGTGTAGGGAAAGAGTGT | AGCAAGATCGGAAGAGCGTCGTGTAGGGAAAGAGTGT |
| 3 | CATA | P1_03.1 | ACACTCTTTCCCTACACGACGCTCTTCCGATCTCATATGCA | P1_03.2 | TATGAGATCGGAAGAGCGTCGTGTAGGGAAAGAGTGT | TATGAGATCGGAAGAGCGTCGTGTAGGGAAAGAGTGT |
| 4 | CGAG | P1_04.1 | ACACTCTTTCCCTACACGACGCTCTTCCGATCTCGAGTGCA | P1_04.2 | CTCGAGATCGGAAGAGCGTCGTGTAGGGAAAGAGTGT | CTCGAGATCGGAAGAGCGTCGTGTAGGGAAAGAGTGT |
| 5 | GCTT | P1_05.1 | ACACTCTTTCCCTACACGACGCTCTTCCGATCTGCTTTGCA | P1_05.2 | AAGCAGATCGGAAGAGCGTCGTGTAGGGAAAGAGTGT | AAGCAGATCGGAAGAGCGTCGTGTAGGGAAAGAGTGT |
| 6 | ATCA | P1_06.1 | ACACTCTTTCCCTACACGACGCTCTTCCGATCTATCATGCA | P1_06.2 | TGATAGATCGGAAGAGCGTCGTGTAGGGAAAGAGTGT | TGATAGATCGGAAGAGCGTCGTGTAGGGAAAGAGTGT |
| 7 | GACG | P1_07.1 | ACACTCTTTCCCTACACGACGCTCTTCCGATCTGACGTGCA | P1_07.2 | CGTCAGATCGGAAGAGCGTCGTGTAGGGAAAGAGTGT | CGTCAGATCGGAAGAGCGTCGTGTAGGGAAAGAGTGT |
| 8 | CTGT | P1_08.1 | ACACTCTTTCCCTACACGACGCTCTTCCGATCTCTGTTGCA | P1_08.2 | ACAGAGATCGGAAGAGCGTCGTGTAGGGAAAGAGTGT | ACAGAGATCGGAAGAGCGTCGTGTAGGGAAAGAGTGT |
| 9 | TCAA | P1_09.1 | ACACTCTTTCCCTACACGACGCTCTTCCGATCTTCAATGCA | P1_09.2 | TTGAAGATCGGAAGAGCGTCGTGTAGGGAAAGAGTGT | TTGAAGATCGGAAGAGCGTCGTGTAGGGAAAGAGTGT |
| 10 | AGTCA | P1_10.1 | ACACTCTTTCCCTACACGACGCTCTTCCGATCTAGTCATGCA | P1_10.2 | TGACTAGATCGGAAGAGCGTCGTGTAGGGAAAGAGTGT | TGACTAGATCGGAAGAGCGTCGTGTAGGGAAAGAGTGT |
| 11 | TCACG | P1_11.1 | ACACTCTTTCCCTACACGACGCTCTTCCGATCTTCACGTGCA | P1_11.2 | CGTGAAGATCGGAAGAGCGTCGTGTAGGGAAAGAGTGT | CGTGAAGATCGGAAGAGCGTCGTGTAGGGAAAGAGTGT |
| 12 | CTGCA | P1_12.1 | ACACTCTTTCCCTACACGACGCTCTTCCGATCTCTGCATGCA | P1_12.2 | TGCAGAGATCGGAAGAGCGTCGTGTAGGGAAAGAGTGT | TGCAGAGATCGGAAGAGCGTCGTGTAGGGAAAGAGTGT |
| 13 | CATCG | P1_13.1 | ACACTCTTTCCCTACACGACGCTCTTCCGATCTCATCGTGCA | P1_13.2 | CGATGAGATCGGAAGAGCGTCGTGTAGGGAAAGAGTGT | CGATGAGATCGGAAGAGCGTCGTGTAGGGAAAGAGTGT |
| 14 | ATCGA | P1_14.1 | ACACTCTTTCCCTACACGACGCTCTTCCGATCTATCGATGCA | P1_14.2 | TCGATAGATCGGAAGAGCGTCGTGTAGGGAAAGAGTGT | TCGATAGATCGGAAGAGCGTCGTGTAGGGAAAGAGTGT |
| 15 | TCGAA | P1_15.1 | ACACTCTTTCCCTACACGACGCTCTTCCGATCTTCGAATGCA | P1_15.2 | TTCGAAGATCGGAAGAGCGTCGTGTAGGGAAAGAGTGT | TTCGAAGATCGGAAGAGCGTCGTGTAGGGAAAGAGTGT |
| 16 | ACCTG | P1_16.1 | ACACTCTTTCCCTACACGACGCTCTTCCGATCTACCTGTGCA | P1_16.2 | CAGGTAGATCGGAAGAGCGTCGTGTAGGGAAAGAGTGT | CAGGTAGATCGGAAGAGCGTCGTGTAGGGAAAGAGTGT |
| 17 | CTCAG | P1_17.1 | ACACTCTTTCCCTACACGACGCTCTTCCGATCTCTCAGTGCA | P1_17.2 | CTGAGAGATCGGAAGAGCGTCGTGTAGGGAAAGAGTGT | CTGAGAGATCGGAAGAGCGTCGTGTAGGGAAAGAGTGT |
| 18 | CGCTA | P1_18.1 | ACACTCTTTCCCTACACGACGCTCTTCCGATCTCGCTATGCA | P1_18.2 | TAGCGAGATCGGAAGAGCGTCGTGTAGGGAAAGAGTGT | TAGCGAGATCGGAAGAGCGTCGTGTAGGGAAAGAGTGT |
| 19 | CCTGA | P1_19.1 | ACACTCTTTCCCTACACGACGCTCTTCCGATCTCCTGATGCA | P1_19.2 | TCAGGAGATCGGAAGAGCGTCGTGTAGGGAAAGAGTGT | TCAGGAGATCGGAAGAGCGTCGTGTAGGGAAAGAGTGT |
| 20 | CGACT | P1_20.1 | ACACTCTTTCCCTACACGACGCTCTTCCGATCTCGACTTGCA | P1_20.2 | AGTCGAGATCGGAAGAGCGTCGTGTAGGGAAAGAGTGT | AGTCGAGATCGGAAGAGCGTCGTGTAGGGAAAGAGTGT |
| 21 | ACGCT | P1_21.1 | ACACTCTTTCCCTACACGACGCTCTTCCGATCTACGCTTGCA | P1_21.2 | AGCGTAGATCGGAAGAGCGTCGTGTAGGGAAAGAGTGT | AGCGTAGATCGGAAGAGCGTCGTGTAGGGAAAGAGTGT |
| 22 | GCCAT | P1_22.1 | ACACTCTTTCCCTACACGACGCTCTTCCGATCTGCCATTGCA | P1_22.2 | ATGGCAGATCGGAAGAGCGTCGTGTAGGGAAAGAGTGT | ATGGCAGATCGGAAGAGCGTCGTGTAGGGAAAGAGTGT |
| 23 | CACGT | P1_23.1 | ACACTCTTTCCCTACACGACGCTCTTCCGATCTCACGTTGCA | P1_23.2 | ACGTGAGATCGGAAGAGCGTCGTGTAGGGAAAGAGTGT | ACGTGAGATCGGAAGAGCGTCGTGTAGGGAAAGAGTGT |
| 24 | GTTCCA | P1_24.1 | ACACTCTTTCCCTACACGACGCTCTTCCGATCTGTTCCATGCA | P1_24.2 | TGGAACAGATCGGAAGAGCGTCGTGTAGGGAAAGAGTGT | TGGAACAGATCGGAAGAGCGTCGTGTAGGGAAAGAGTGT |
| 25 | TGTGCA | P1_25.1 | ACACTCTTTCCCTACACGACGCTCTTCCGATCTTGTGCATGCA | P1_25.2 | TGCACAAGATCGGAAGAGCGTCGTGTAGGGAAAGAGTGT | TGCACAAGATCGGAAGAGCGTCGTGTAGGGAAAGAGTGT |
| 26 | TTGACA | P1_26.1 | ACACTCTTTCCCTACACGACGCTCTTCCGATCTTTGACATGCA | P1_26.2 | TGTCAAAGATCGGAAGAGCGTCGTGTAGGGAAAGAGTGT | TGTCAAAGATCGGAAGAGCGTCGTGTAGGGAAAGAGTGT |
| 27 | AGCTGA | P1_27.1 | ACACTCTTTCCCTACACGACGCTCTTCCGATCTAGCTGATGCA | P1_27.2 | TCAGCTAGATCGGAAGAGCGTCGTGTAGGGAAAGAGTGT | TCAGCTAGATCGGAAGAGCGTCGTGTAGGGAAAGAGTGT |
| 28 | TGGCAA | P1_28.1 | ACACTCTTTCCCTACACGACGCTCTTCCGATCTTGGCAATGCA | P1_28.2 | TTGCCAAGATCGGAAGAGCGTCGTGTAGGGAAAGAGTGT | TTGCCAAGATCGGAAGAGCGTCGTGTAGGGAAAGAGTGT |
| 29 | CTATCG | P1_29.1 | ACACTCTTTCCCTACACGACGCTCTTCCGATCTCTATCGTGCA | P1_29.2 | CGATAGAGATCGGAAGAGCGTCGTGTAGGGAAAGAGTGT | CGATAGAGATCGGAAGAGCGTCGTGTAGGGAAAGAGTGT |
| 30 | GCTGAA | P1_30.1 | ACACTCTTTCCCTACACGACGCTCTTCCGATCTGCTGAATGCA | P1_30.2 | TTCAGCAGATCGGAAGAGCGTCGTGTAGGGAAAGAGTGT | TTCAGCAGATCGGAAGAGCGTCGTGTAGGGAAAGAGTGT |
| 31 | TTCCGA | P1_31.1 | ACACTCTTTCCCTACACGACGCTCTTCCGATCTTTCCGATGCA | P1_31.2 | TCGGAAAGATCGGAAGAGCGTCGTGTAGGGAAAGAGTGT | TCGGAAAGATCGGAAGAGCGTCGTGTAGGGAAAGAGTGT |
| 32 | GACTCT | P1_32.1 | ACACTCTTTCCCTACACGACGCTCTTCCGATCTGACTCTTGCA | P1_32.2 | AGAGTCAGATCGGAAGAGCGTCGTGTAGGGAAAGAGTGT | AGAGTCAGATCGGAAGAGCGTCGTGTAGGGAAAGAGTGT |
| 33 | ATGGCG | P1_33.1 | ACACTCTTTCCCTACACGACGCTCTTCCGATCTATGGCGTGCA | P1_33.2 | CGCCATAGATCGGAAGAGCGTCGTGTAGGGAAAGAGTGT | CGCCATAGATCGGAAGAGCGTCGTGTAGGGAAAGAGTGT |
| 34 | TCATGG | P1_34.1 | ACACTCTTTCCCTACACGACGCTCTTCCGATCTTCATGGTGCA | P1_34.2 | CCATGAAGATCGGAAGAGCGTCGTGTAGGGAAAGAGTGT | CCATGAAGATCGGAAGAGCGTCGTGTAGGGAAAGAGTGT |
| 35 | CATCCG | P1_35.1 | ACACTCTTTCCCTACACGACGCTCTTCCGATCTCATCCGTGCA | P1_35.2 | CGGATGAGATCGGAAGAGCGTCGTGTAGGGAAAGAGTGT | CGGATGAGATCGGAAGAGCGTCGTGTAGGGAAAGAGTGT |
| 36 | CCGTCA | P1_36.1 | ACACTCTTTCCCTACACGACGCTCTTCCGATCTCCGTCATGCA | P1_36.2 | TGACGGAGATCGGAAGAGCGTCGTGTAGGGAAAGAGTGT | TGACGGAGATCGGAAGAGCGTCGTGTAGGGAAAGAGTGT |
| 37 | GTACGT | P1_37.1 | ACACTCTTTCCCTACACGACGCTCTTCCGATCTGTACGTTGCA | P1_37.2 | ACGTACAGATCGGAAGAGCGTCGTGTAGGGAAAGAGTGT | ACGTACAGATCGGAAGAGCGTCGTGTAGGGAAAGAGTGT |
| 38 | TAGGCT | P1_38.1 | ACACTCTTTCCCTACACGACGCTCTTCCGATCTTAGGCTTGCA | P1_38.2 | AGCCTAAGATCGGAAGAGCGTCGTGTAGGGAAAGAGTGT | AGCCTAAGATCGGAAGAGCGTCGTGTAGGGAAAGAGTGT |
| 39 | GGCTAG | P1_39.1 | ACACTCTTTCCCTACACGACGCTCTTCCGATCTGGCTAGTGCA | P1_39.2 | CTAGCCAGATCGGAAGAGCGTCGTGTAGGGAAAGAGTGT | CTAGCCAGATCGGAAGAGCGTCGTGTAGGGAAAGAGTGT |
| 40 | CATGTA | P1_40.1 | ACACTCTTTCCCTACACGACGCTCTTCCGATCTCATGTATGCA | P1_40.2 | TACATGAGATCGGAAGAGCGTCGTGTAGGGAAAGAGTGT | TACATGAGATCGGAAGAGCGTCGTGTAGGGAAAGAGTGT |
| 41 | ATTCGG | P1_41.1 | ACACTCTTTCCCTACACGACGCTCTTCCGATCTATTCGGTGCA | P1_41.2 | CCGAATAGATCGGAAGAGCGTCGTGTAGGGAAAGAGTGT | CCGAATAGATCGGAAGAGCGTCGTGTAGGGAAAGAGTGT |
| 42 | TGACCT | P1_42.1 | ACACTCTTTCCCTACACGACGCTCTTCCGATCTTGACCTTGCA | P1_42.2 | AGGTCAAGATCGGAAGAGCGTCGTGTAGGGAAAGAGTGT | AGGTCAAGATCGGAAGAGCGTCGTGTAGGGAAAGAGTGT |
| 43 | GCTACT | P1_43.1 | ACACTCTTTCCCTACACGACGCTCTTCCGATCTGCTACTTGCA | P1_43.2 | AGTAGCAGATCGGAAGAGCGTCGTGTAGGGAAAGAGTGT | AGTAGCAGATCGGAAGAGCGTCGTGTAGGGAAAGAGTGT |
| 44 | TCGGTA | P1_44.1 | ACACTCTTTCCCTACACGACGCTCTTCCGATCTTCGGTATGCA | P1_44.2 | TACCGAAGATCGGAAGAGCGTCGTGTAGGGAAAGAGTGT | TACCGAAGATCGGAAGAGCGTCGTGTAGGGAAAGAGTGT |
| 45 | CTGAGG | P1_45.1 | ACACTCTTTCCCTACACGACGCTCTTCCGATCTCTGAGGTGCA | P1_45.2 | CCTCAGAGATCGGAAGAGCGTCGTGTAGGGAAAGAGTGT | CCTCAGAGATCGGAAGAGCGTCGTGTAGGGAAAGAGTGT |
| 46 | GCCTTA | P1_46.1 | ACACTCTTTCCCTACACGACGCTCTTCCGATCTGCCTTATGCA | P1_46.2 | TAAGGCAGATCGGAAGAGCGTCGTGTAGGGAAAGAGTGT | TAAGGCAGATCGGAAGAGCGTCGTGTAGGGAAAGAGTGT |
| 47 | CGATGT | P1_47.1 | ACACTCTTTCCCTACACGACGCTCTTCCGATCTCGATGTTGCA | P1_47.2 | ACATCGAGATCGGAAGAGCGTCGTGTAGGGAAAGAGTGT | ACATCGAGATCGGAAGAGCGTCGTGTAGGGAAAGAGTGT |
| 48 | GATTACA | P1_48.1 | ACACTCTTTCCCTACACGACGCTCTTCCGATCTGATTACATGCA | P1_48.2 | TGTAATCAGATCGGAAGAGCGTCGTGTAGGGAAAGAGTGT | TGTAATCAGATCGGAAGAGCGTCGTGTAGGGAAAGAGTGT |
| 49 | GGTAGCA | P1_49.1 | ACACTCTTTCCCTACACGACGCTCTTCCGATCTGGTAGCATGCA | P1_49.2 | TGCTACCAGATCGGAAGAGCGTCGTGTAGGGAAAGAGTGT | TGCTACCAGATCGGAAGAGCGTCGTGTAGGGAAAGAGTGT |
| 50 | GTGACCA | P1_50.1 | ACACTCTTTCCCTACACGACGCTCTTCCGATCTGTGACCATGCA | P1_50.2 | TGGTCACAGATCGGAAGAGCGTCGTGTAGGGAAAGAGTGT | TGGTCACAGATCGGAAGAGCGTCGTGTAGGGAAAGAGTGT |
| 51 | TTATGCA | P1_51.1 | ACACTCTTTCCCTACACGACGCTCTTCCGATCTTTATGCATGCA | P1_51.2 | TGCATAAAGATCGGAAGAGCGTCGTGTAGGGAAAGAGTGT | TGCATAAAGATCGGAAGAGCGTCGTGTAGGGAAAGAGTGT |
| 52 | ATTGGCA | P1_52.1 | ACACTCTTTCCCTACACGACGCTCTTCCGATCTATTGGCATGCA | P1_52.2 | TGCCAATAGATCGGAAGAGCGTCGTGTAGGGAAAGAGTGT | TGCCAATAGATCGGAAGAGCGTCGTGTAGGGAAAGAGTGT |
| 53 | TGGTACA | P1_53.1 | ACACTCTTTCCCTACACGACGCTCTTCCGATCTTGGTACATGCA | P1_53.2 | TGTACCAAGATCGGAAGAGCGTCGTGTAGGGAAAGAGTGT | TGTACCAAGATCGGAAGAGCGTCGTGTAGGGAAAGAGTGT |
| 54 | GACCTCA | P1_54.1 | ACACTCTTTCCCTACACGACGCTCTTCCGATCTGACCTCATGCA | P1_54.2 | TGAGGTCAGATCGGAAGAGCGTCGTGTAGGGAAAGAGTGT | TGAGGTCAGATCGGAAGAGCGTCGTGTAGGGAAAGAGTGT |
| 55 | TGTGCCA | P1_55.1 | ACACTCTTTCCCTACACGACGCTCTTCCGATCTTGTGCCATGCA | P1_55.2 | TGGCACAAGATCGGAAGAGCGTCGTGTAGGGAAAGAGTGT | TGGCACAAGATCGGAAGAGCGTCGTGTAGGGAAAGAGTGT |
| 56 | TAGACCG | P1_56.1 | ACACTCTTTCCCTACACGACGCTCTTCCGATCTTAGACCGTGCA | P1_56.2 | CGGTCTAAGATCGGAAGAGCGTCGTGTAGGGAAAGAGTGT | CGGTCTAAGATCGGAAGAGCGTCGTGTAGGGAAAGAGTGT |
| 57 | GGATTCA | P1_57.1 | ACACTCTTTCCCTACACGACGCTCTTCCGATCTGGATTCATGCA | P1_57.2 | TGAATCCAGATCGGAAGAGCGTCGTGTAGGGAAAGAGTGT | TGAATCCAGATCGGAAGAGCGTCGTGTAGGGAAAGAGTGT |
| 58 | GATCCAA | P1_58.1 | ACACTCTTTCCCTACACGACGCTCTTCCGATCTGATCCAATGCA | P1_58.2 | TTGGATCAGATCGGAAGAGCGTCGTGTAGGGAAAGAGTGT | TTGGATCAGATCGGAAGAGCGTCGTGTAGGGAAAGAGTGT |
| 59 | CTGGACA | P1_59.1 | ACACTCTTTCCCTACACGACGCTCTTCCGATCTCTGGACATGCA | P1_59.2 | TGTCCAGAGATCGGAAGAGCGTCGTGTAGGGAAAGAGTGT | TGTCCAGAGATCGGAAGAGCGTCGTGTAGGGAAAGAGTGT |
| 60 | AGACTCG | P1_60.1 | ACACTCTTTCCCTACACGACGCTCTTCCGATCTAGACTCGTGCA | P1_60.2 | CGAGTCTAGATCGGAAGAGCGTCGTGTAGGGAAAGAGTGT | CGAGTCTAGATCGGAAGAGCGTCGTGTAGGGAAAGAGTGT |
| 61 | AATTGCG | P1_61.1 | ACACTCTTTCCCTACACGACGCTCTTCCGATCTAATTGCGTGCA | P1_61.2 | CGCAATTAGATCGGAAGAGCGTCGTGTAGGGAAAGAGTGT | CGCAATTAGATCGGAAGAGCGTCGTGTAGGGAAAGAGTGT |
| 62 | TCCAGGA | P1_62.1 | ACACTCTTTCCCTACACGACGCTCTTCCGATCTTCCAGGATGCA | P1_62.2 | TCCTGGAAGATCGGAAGAGCGTCGTGTAGGGAAAGAGTGT | TCCTGGAAGATCGGAAGAGCGTCGTGTAGGGAAAGAGTGT |
| 63 | TCAGCAG | P1_63.1 | ACACTCTTTCCCTACACGACGCTCTTCCGATCTTCAGCAGTGCA | P1_63.2 | CTGCTGAAGATCGGAAGAGCGTCGTGTAGGGAAAGAGTGT | CTGCTGAAGATCGGAAGAGCGTCGTGTAGGGAAAGAGTGT |
| 64 | CAGTGCA | P1_64.1 | ACACTCTTTCCCTACACGACGCTCTTCCGATCTCAGTGCATGCA | P1_64.2 | TGCACTGAGATCGGAAGAGCGTCGTGTAGGGAAAGAGTGT | TGCACTGAGATCGGAAGAGCGTCGTGTAGGGAAAGAGTGT |
| 65 | GTACCGA | P1_65.1 | ACACTCTTTCCCTACACGACGCTCTTCCGATCTGTACCGATGCA | P1_65.2 | TCGGTACAGATCGGAAGAGCGTCGTGTAGGGAAAGAGTGT | TCGGTACAGATCGGAAGAGCGTCGTGTAGGGAAAGAGTGT |
| 66 | TGTAACG | P1_66.1 | ACACTCTTTCCCTACACGACGCTCTTCCGATCTTGTAACGTGCA | P1_66.2 | CGTTACAAGATCGGAAGAGCGTCGTGTAGGGAAAGAGTGT | CGTTACAAGATCGGAAGAGCGTCGTGTAGGGAAAGAGTGT |
| 67 | TACGATA | P1_67.1 | ACACTCTTTCCCTACACGACGCTCTTCCGATCTTACGATATGCA | P1_67.2 | TATCGTAAGATCGGAAGAGCGTCGTGTAGGGAAAGAGTGT | TATCGTAAGATCGGAAGAGCGTCGTGTAGGGAAAGAGTGT |
| 68 | GTAAGCG | P1_68.1 | ACACTCTTTCCCTACACGACGCTCTTCCGATCTGTAAGCGTGCA | P1_68.2 | CGCTTACAGATCGGAAGAGCGTCGTGTAGGGAAAGAGTGT | CGCTTACAGATCGGAAGAGCGTCGTGTAGGGAAAGAGTGT |
| 69 | ATGCAAT | P1_69.1 | ACACTCTTTCCCTACACGACGCTCTTCCGATCTATGCAATTGCA | P1_69.2 | ATTGCATAGATCGGAAGAGCGTCGTGTAGGGAAAGAGTGT | ATTGCATAGATCGGAAGAGCGTCGTGTAGGGAAAGAGTGT |
| 70 | CCGGTAA | P1_70.1 | ACACTCTTTCCCTACACGACGCTCTTCCGATCTCCGGTAATGCA | P1_70.2 | TTACCGGAGATCGGAAGAGCGTCGTGTAGGGAAAGAGTGT | TTACCGGAGATCGGAAGAGCGTCGTGTAGGGAAAGAGTGT |
| 71 | AGCTCCG | P1_71.1 | ACACTCTTTCCCTACACGACGCTCTTCCGATCTAGCTCCGTGCA | P1_71.2 | CGGAGCTAGATCGGAAGAGCGTCGTGTAGGGAAAGAGTGT | CGGAGCTAGATCGGAAGAGCGTCGTGTAGGGAAAGAGTGT |
| 72 | AACTCG | P1_72.1 | ACACTCTTTCCCTACACGACGCTCTTCCGATCTAACTCGTGCA | P1_72.2 | CGAGTTAGATCGGAAGAGCGTCGTGTAGGGAAAGAGTGT | CGAGTTAGATCGGAAGAGCGTCGTGTAGGGAAAGAGTGT |
| 73 | AGAATGCA | P1_73.1 | ACACTCTTTCCCTACACGACGCTCTTCCGATCTAGAATGCATGCA | P1_73.2 | TGCATTCTAGATCGGAAGAGCGTCGTGTAGGGAAAGAGTGT | TGCATTCTAGATCGGAAGAGCGTCGTGTAGGGAAAGAGTGT |
| 74 | GAATAGCA | P1_74.1 | ACACTCTTTCCCTACACGACGCTCTTCCGATCTGAATAGCATGCA | P1_74.2 | TGCTATTCAGATCGGAAGAGCGTCGTGTAGGGAAAGAGTGT | TGCTATTCAGATCGGAAGAGCGTCGTGTAGGGAAAGAGTGT |
| 75 | ATGAGACA | P1_75.1 | ACACTCTTTCCCTACACGACGCTCTTCCGATCTATGAGACATGCA | P1_75.2 | TGTCTCATAGATCGGAAGAGCGTCGTGTAGGGAAAGAGTGT | TGTCTCATAGATCGGAAGAGCGTCGTGTAGGGAAAGAGTGT |
| 76 | TGCCACCA | P1_76.1 | ACACTCTTTCCCTACACGACGCTCTTCCGATCTTGCCACCATGCA | P1_76.2 | TGGTGGCAAGATCGGAAGAGCGTCGTGTAGGGAAAGAGTGT | TGGTGGCAAGATCGGAAGAGCGTCGTGTAGGGAAAGAGTGT |
| 77 | ATAGAGCA | P1_77.1 | ACACTCTTTCCCTACACGACGCTCTTCCGATCTATAGAGCATGCA | P1_77.2 | TGCTCTATAGATCGGAAGAGCGTCGTGTAGGGAAAGAGTGT | TGCTCTATAGATCGGAAGAGCGTCGTGTAGGGAAAGAGTGT |
| 78 | ACTCGCCA | P1_78.1 | ACACTCTTTCCCTACACGACGCTCTTCCGATCTACTCGCCATGCA | P1_78.2 | TGGCGAGTAGATCGGAAGAGCGTCGTGTAGGGAAAGAGTGT | TGGCGAGTAGATCGGAAGAGCGTCGTGTAGGGAAAGAGTGT |
| 79 | TAGGAACA | P1_79.1 | ACACTCTTTCCCTACACGACGCTCTTCCGATCTTAGGAACATGCA | P1_79.2 | TGTTCCTAAGATCGGAAGAGCGTCGTGTAGGGAAAGAGTGT | TGTTCCTAAGATCGGAAGAGCGTCGTGTAGGGAAAGAGTGT |
| 80 | GATACGAA | P1_80.1 | ACACTCTTTCCCTACACGACGCTCTTCCGATCTGATACGAATGCA | P1_80.2 | TTCGTATCAGATCGGAAGAGCGTCGTGTAGGGAAAGAGTGT | TTCGTATCAGATCGGAAGAGCGTCGTGTAGGGAAAGAGTGT |
| 81 | GCACCTCA | P1_81.1 | ACACTCTTTCCCTACACGACGCTCTTCCGATCTGCACCTCATGCA | P1_81.2 | TGAGGTGCAGATCGGAAGAGCGTCGTGTAGGGAAAGAGTGT | TGAGGTGCAGATCGGAAGAGCGTCGTGTAGGGAAAGAGTGT |
| 82 | CACTGCCA | P1_82.1 | ACACTCTTTCCCTACACGACGCTCTTCCGATCTCACTGCCATGCA | P1_82.2 | TGGCAGTGAGATCGGAAGAGCGTCGTGTAGGGAAAGAGTGT | TGGCAGTGAGATCGGAAGAGCGTCGTGTAGGGAAAGAGTGT |
| 83 | ACGATGAA | P1_83.1 | ACACTCTTTCCCTACACGACGCTCTTCCGATCTACGATGAATGCA | P1_83.2 | TTCATCGTAGATCGGAAGAGCGTCGTGTAGGGAAAGAGTGT | TTCATCGTAGATCGGAAGAGCGTCGTGTAGGGAAAGAGTGT |
| 84 | CGCACACT | P1_84.1 | ACACTCTTTCCCTACACGACGCTCTTCCGATCTCGCACACTTGCA | P1_84.2 | AGTGTGCGAGATCGGAAGAGCGTCGTGTAGGGAAAGAGTGT | AGTGTGCGAGATCGGAAGAGCGTCGTGTAGGGAAAGAGTGT |
| 85 | GGTCTT | P1_85.1 | ACACTCTTTCCCTACACGACGCTCTTCCGATCTGGTCTTTGCA | P1_85.2 | AAGACCAGATCGGAAGAGCGTCGTGTAGGGAAAGAGTGT | AAGACCAGATCGGAAGAGCGTCGTGTAGGGAAAGAGTGT |
| 86 | CAAGTAGA | P1_86.1 | ACACTCTTTCCCTACACGACGCTCTTCCGATCTCAAGTAGATGCA | P1_86.2 | TCTACTTGAGATCGGAAGAGCGTCGTGTAGGGAAAGAGTGT | TCTACTTGAGATCGGAAGAGCGTCGTGTAGGGAAAGAGTGT |
| 87 | GCAAGAAT | P1_87.1 | ACACTCTTTCCCTACACGACGCTCTTCCGATCTGCAAGAATTGCA | P1_87.2 | ATTCTTGCAGATCGGAAGAGCGTCGTGTAGGGAAAGAGTGT | ATTCTTGCAGATCGGAAGAGCGTCGTGTAGGGAAAGAGTGT |
| 88 | ACCTACCG | P1_88.1 | ACACTCTTTCCCTACACGACGCTCTTCCGATCTACCTACCGTGCA | P1_88.2 | CGGTAGGTAGATCGGAAGAGCGTCGTGTAGGGAAAGAGTGT | CGGTAGGTAGATCGGAAGAGCGTCGTGTAGGGAAAGAGTGT |
| 89 | CTACCACG | P1_89.1 | ACACTCTTTCCCTACACGACGCTCTTCCGATCTCTACCACGTGCA | P1_89.2 | CGTGGTAGAGATCGGAAGAGCGTCGTGTAGGGAAAGAGTGT | CGTGGTAGAGATCGGAAGAGCGTCGTGTAGGGAAAGAGTGT |
| 90 | TAGAACGA | P1_90.1 | ACACTCTTTCCCTACACGACGCTCTTCCGATCTTAGAACGATGCA | P1_90.2 | TCGTTCTAAGATCGGAAGAGCGTCGTGTAGGGAAAGAGTGT | TCGTTCTAAGATCGGAAGAGCGTCGTGTAGGGAAAGAGTGT |
| 91 | AGCAGTAA | P1_91.1 | ACACTCTTTCCCTACACGACGCTCTTCCGATCTAGCAGTAATGCA | P1_91.2 | TTACTGCTAGATCGGAAGAGCGTCGTGTAGGGAAAGAGTGT | TTACTGCTAGATCGGAAGAGCGTCGTGTAGGGAAAGAGTGT |
| 92 | GAACTGAA | P1_92.1 | ACACTCTTTCCCTACACGACGCTCTTCCGATCTGAACTGAATGCA | P1_92.2 | TTCAGTTCAGATCGGAAGAGCGTCGTGTAGGGAAAGAGTGT | TTCAGTTCAGATCGGAAGAGCGTCGTGTAGGGAAAGAGTGT |
| 93 | ACTCCACG | P1_93.1 | ACACTCTTTCCCTACACGACGCTCTTCCGATCTACTCCACGTGCA | P1_93.2 | CGTGGAGTAGATCGGAAGAGCGTCGTGTAGGGAAAGAGTGT | CGTGGAGTAGATCGGAAGAGCGTCGTGTAGGGAAAGAGTGT |
| 94 | GAAGACAT | P1_94.1 | ACACTCTTTCCCTACACGACGCTCTTCCGATCTGAAGACATTGCA | P1_94.2 | ATGTCTTCAGATCGGAAGAGCGTCGTGTAGGGAAAGAGTGT | ATGTCTTCAGATCGGAAGAGCGTCGTGTAGGGAAAGAGTGT |
| 95 | CGGTATGT | P1_95.1 | ACACTCTTTCCCTACACGACGCTCTTCCGATCTCGGTATGTTGCA | P1_95.2 | ACATACCGAGATCGGAAGAGCGTCGTGTAGGGAAAGAGTGT | ACATACCGAGATCGGAAGAGCGTCGTGTAGGGAAAGAGTGT |
| 96 | TCCGCACA | P1_96.1 | ACACTCTTTCCCTACACGACGCTCTTCCGATCTTCCGCACATGCA | P1_96.2 | TGTGCGGAAGATCGGAAGAGCGTCGTGTAGGGAAAGAGTGT | TGTGCGGAAGATCGGAAGAGCGTCGTGTAGGGAAAGAGTGT |

1. **Double restriction digest**
   1. Prepare master mix I (see below, 3 µL prepared per sample), mix and centrifuge.

| **Master Mix I: Digestion** | Vol 1x |
| --- | --- |
| 10x T4 buffer | 0.9 |
| 1M NaCl | 0.45 |
| 1mg/mL BSA | 0.45 |
| H2O | 0.85 |
| MseI(10,000U/ml) | 0.1 |
| SbfI(HF)(20,000U/ml) | 0.25 |
| Total | 3 |

- 1. Place 6 µL of sample DNA in each well of a plate.
  2. Add 3 µL of the combined master mix I to each well. The total reaction volume should be 9 µL.
  3. Cover and seal the plate, centrifuge and incubate at 37°C for 5 hours on a thermal cycler with a heated lid. Heat kill the enzyme (20 mins at 65ºC). Keep at 4ºC afterwards.

1. **Adaptor Ligation**
   1. Thaw P1 and P2 adaptors. These adaptors should already be annealed (step 0).
   2. Prepare master mix II (see below, 1.6 µL prepared per sample), mix well. As above, it is best to prepare an extra 20% (1.2x/sample).

| **MASTER MIXII: LIGATION** | Vol 1x |
| --- | --- |
| 10x T4 Buffer | 0.16 |
| 1M NaCl | 0.13 |
| 1 mg/ml BSA | 0.13 |
| H20 | 0.0125 |
| P2 (MseI) adapter 10uM | 1 |
| T4 DNA Ligase (400,000U/ml) | 0.1675 |
| Total | 1.6 |

- 1. Add 1.6 µL to each well of the restriction digested DNA.
  2. Add 1 µL of the P1 (SbfI) adaptor to each well (a unique barcoded adaptor for each DNA sample)
  3. The total reaction volume should now be 11.6 µL. Cover and seal the plate, vortex gently, centrifuge and incubate at 16° C for 3 hours on a thermocycler.
  4. Dilute the Restriction-Ligation reaction up to 40 µL withTris 10 mM (=EB) (or 0.1x TE for long-term storage). Store at 4° C for up to one month, or -20° C for longer periods.

1. **III. Purification**

*AMPure* purification with ratio of 0.7x Resuspend in 40 µL EB

1. **PCR Amplification**

This PCR step uses the Illumina PCR primers to amplify fragments that have our adapters + barcodes ligated onto the ends. To compensate for stochastic differences in PCR production of fragments in reactions, we run the PCR reaction twice.

- 1. Prepare master mix III, (see below, 8 µL per sample, but preparing 2 PCR reactions per sample), vortex and centrifuge. If only 2 index primers will be used, use the ILLPCR2_ind06 and ILLPCR2_ind12; if three primers, use 4, 6, 12; if six primers use 2,4,5,6,7,12.

| **MASTER MIX III: PCR** | vol 1x |
| --- | --- |
| Water | 4.15 |
| Q5 Buffer | 2 |
| dNTP (25mM) | 0.08 |
| PCR Primer Mix | 0.67 |
| Q5 Taq | 0.1 |
| GC enhancer | 1 |
| Total | 8 |

- 1. Add 8 µL of the combined master mix III to each well of a plate.
  2. Add 2 µL of the diluted ligation product from step 2 or of the purification product if step 3 was performed.
  3. Thermal cycler profile for this PCR: 98° C for 30s; 15 cycles of: 98° C for 20s, 60° C for 30s, 72° C for 40s; final extension at 72° C for 10 min.
  4. Prepare master mix IV (see below, 1 µL per sample)

| **MASTER MIX IV: PCR final cycle** | vol 1x |
| --- | --- |
| Water | 0.385 |
| Q5 Buffer | 0.2 |
| PCR primer mix | 0.335 |
| dNTP (25 mM) | 0.08 |
| Total | 1 |

6. Add 1 µL to each PCR product (keep cold), run thermocycler profile as follows: 98° C for 3 min, 60° C for 2 min, 72° C for 12 min.

1. **Pool replicates and verify amplification success**
   1. Pool the two replicates together -> final volume of 22 µL /well
   2. For each well verify amplification success through electrophoresis
2. **AMPure purification, quantification and standardize concentration**
   1. Purify each well of the plates using *AMPure* ratio 0.7x. Re-elute in 20 µLEB.
   2. Quantify with *Picogreen* the concentration of each well of the plates.
   3. Standardize concentration according to the median concentration of samples. Lower concentrations are left unaltered.
3. **Pool samples per plate, quantify and standardize concentrations**
   1. Pool 16 µL of each well per plate together (1.5mL)
   2. Quantify concentration using *Q bit*
   3. Bring pools to equal molarity
   4. Pool the different PCR plate pools together
   5. Re-concentrate with *SPEEDVAX*
4. **Size selection**
   1. Analyze library profile with Fragment Analyzer (FA) in order to determine the optimal size to select
   2. Size select using *Blue Pippin* 2%
   3. Verify size selection success with FA

**Sequence with Illumina Hiseq single end (100bp**
